# Guidelines for the management of emergencies and critical illness in pediatric and adult patients with sickle cell disease

**DOI:** 10.1186/s13613-025-01479-3

**Published:** 2025-05-29

**Authors:** Armand Mekontso Dessap, Stephane Dauger, Mehdi Khellaf, Maite Agbakou, Sophie Agut, François Angoulvant, Jean-Benoît Arlet, Cécile Aubron, Florent Baudin, Florence Boissier, Nicolas Bounaud, Pierre Catoire, Jérôme Cecchini, Djamila Chaiba, Anthony Chauvin, Richard Chocron, Benedicte Douay, Delphine Douillet, Narcisse Elenga, Olivier Flechelle, Ségolène Gendreau, Sybille Goddet, Jeremy Guenezan, Anoosha Habibi, Claire Heilbronner, Bérengère Koehl, Pierrick Le Borgne, Philippe Le Conte, Annick Legras, Michael Levy, Bernard Maitre, Mathieu Oberlin, Mehdi Oualha, Nicolas Peschanski, France Pirenne, Corinne Pondarre, Jérôme Rambaud, Keyvan Razazi, Geoffroy Rousseau, Aurélie Schirmann, Isabelle Thuret, Ruddy Valentino, Guillaume Voiriot, Barbara Villoing, Marion Grimaud, Sandrine Jean

**Affiliations:** 1 AP-HP, Hôpitaux Universitaires Henri-Mondor, Service de Médecine Intensive Réanimation & Centre National de Reference des Syndromes Drépanocytaires, 94010 Créteil, France; 2 Université Paris Est Créteil, INSERM, IMRB, CARMAS, 94010 Créteil, France; 3 AP-HP, Hôpital Universitaire Robert-Debré, Service de Médecine Intensive Réanimation Pédiatrique, 75019 Paris, France; 4 Université Paris Cité, Inserm, NeuroDiderot, 75019 Paris, France; 5 AP-HP, Hôpitaux Universitaires Henri-Mondor, Service d’Accueil des Urgences et Département d’Aval des Urgences, 94010 Créteil, France; 6 CHU Nantes, Médecine Intensive Réanimation, Nantes, France; 7 AP-HP, Hôpital Tenon, Service d’Accueil des Urgences, 75020 Paris, France; 8 AP-HP, Hôpital Robert Debré, Service de Pédiatrie Générale, Paris, France; 9 AP-HP, Hôpital Européen Georges Pompidou, Service de médecine interne, Centre National de Référence des syndromes drépanocytaires majeurs de l’adulte, 75015 Paris, France; 10 Université Paris Cité, 75006 Paris, France; 11 CHU de Brest, Université de Bretagne Occidentale, Service de médecine intensive réanimation, Brest, France; 12 Hospices Civils de Lyon, Hôpital Femme Mère Enfant, Service de réanimation pédiatrique, 69500 Bron, France; 13 CHU de Poitiers, service de Médecine Intensive Réanimation, Poitiers, France; 14 CH Montauban, Service des Urgences, Montauban, France; 15 Improving Emergency Care (IMPEC) FHU, Sorbonne Université, Paris, France; 16 Centre Hospitalier Intercommunal de Créteil, Service de médecine intensive réanimation, 94010 Créteil, France; 17 Hôpital Simone Veil, Service des urgences médico-chirurgicales, Eaubonne, France; 18 AP-HP, Hôpital Lariboisière, Service d’Accueil des Urgences et SMUR, Paris, France; 19 AP-HP, Hôpital Européen Georges Pompidou, Service d’Accueil des Urgences, Paris, France; 20 AP-HP, Hôpital Beaujon, SMUR, Clichy, France; 21 CHU d’Angers, Département de Médecine d’Urgence, Univ Angers, Equipe CARE, Angers, France; 22 Centre Hospitalier de Cayenne, Service de Pédiatrie & Centre de Reference de La Drépanocytose, 97306 Cayenne, France; 23 CHU Martinique, Réanimation pédiatrique et néonatale, Fort de France, France; 24 CHU Dijon, Département universitaire de médecine d’urgences, 21000 Dijon, France; 25 CHU de Poitiers, service des Urgences, Poitiers, France; 26 AP-HP, Hôpitaux Universitaires Henri-Mondor, Service de Médecine Interne, Unité des Maladies Génétiques du globule Rouge & Centre National de Reference des Syndromes Drépanocytaires, 94010 Créteil, France; 27 Université Paris Est Créteil, INSERM, IMRB, Equipe Transfusion et maladies du globule rouge, 94010 Créteil, France; 28 AP-HP, Hôpital Necker, Service de Réanimation et Soins Continus Pédiatriques polyvalents, 75015 Paris, France; 29 AP-HP, Hôpital Robert Debré, Service d’hématologie clinique & Centre National de Référence des syndromes drépanocytaires majeurs de l’enfant, Université Paris Cité, Inserm U1134, 75019 Paris, France; 30 Hôpitaux Universitaires de Strasbourg, Service des Urgences, 67000 Strasbourg, France; 31 CHU de Nantes, Service des urgences, Université de Nantes, Faculté de médecine, Nantes, France; 32 CHRU Tours, Hôpital Bretonneau, Service de Médecine Intensive Réanimation, 37044 Tours, France; 33 Hôpitaux Universitaires de Strasbourg, Université de Strasbourg, Réanimation Pédiatrique Spécialisée, Strasbourg, France; 34 Centre Hospitalier Intercommunal de Créteil, Service de Pneumologie & Centre de Reference des Syndromes Drépanocytaires, 94010 Créteil, France; 35 Centre Hospitalier de Sélestat, Structure des Urgences, 67600 Sélestat, France; 36 AP-HP Centre, Hôpital Necker, Réanimation-Surveillance Continue Médico-chirurgicales-SMUR Pédiatriques. UMR 1343 Pharmacologie et évaluation des thérapeutiques chez l’enfant et la femme enceinte. Université de Paris Cité, 75006 Paris, France; 37 Centre Hospitalier Universitaire de Rennes, Service des Urgences-SAMU-SAS35-SMUR, 35000 Rennes, France; 38 Université Paris Est Créteil, INSERM U955 et Etablissement Français du Sang, Créteil, France; 39 INSERM U955, IMRB, Université Paris XII, Créteil, France; 40 Centre Hospitalier Intercommunal de Créteil, 40 Avenue de Verdun, 94000 Créteil, France; 41 AP-HP, Sorbonne université, Service de réanimation, pédiatrique et néonatale, hôpital Armand-Trousseau, Paris, France; 42 CHRU Tours, Département de Médecine d’Urgence, Tours, France; 43 Hôpital Foch, Service d’urologie, 92150 Suresnes, France; 44 CHU de Marseille, Hôpital de la Timone, Service d’Hématologie Immunologie Oncologie Pédiatrique, Centre National de Reference des Syndromes Drépanocytaires, Marseille, France; 45 Hôpital Universitaire de Martinique, Service de Médecine Intensive réanimation, 97200 Fort-de-France, France; 46 Sorbonne Université, Assistance Publique – Hôpitaux de Paris, Hôpital Tenon, Service de Médecine Intensive Réanimation; Centre de Recherche Saint-Antoine UMRS_938 INSERM, Team 5PMed (Pulmonary Diseases, Pathogens, Physiopathology, Phenogenomics and Personalized Medicine), Paris, France; 47 AP-HP, Hôpital Cochin, Service d’Accueil des Urgences et SMUR, Paris, France; 48 AP-HP, Hôpital Armand Trousseau, Service de Réanimation et Soins intensifs pediatriques et neonataux, Paris, France

## Abstract

Forty-two questions were evaluated concerning management of emergencies and critical illnesses in paediatric and adult patients with sickle cell disease. The assessment covered the following areas: patient referral, vaso-occlusive crisis, acute chest syndrome, transfusion therapy, and priapism. The patient referral category included guidelines for admission to intensive care unit and management at specialized reference centers. The vaso-occlusive crisis topic encompassed pain management, hydration, incentive spirometry, and target oxygen saturation levels. For acute chest syndrome, the focus areas included imaging techniques such as lung ultrasound, computed tomography scans, and echocardiography; treatment with systemic corticosteroids; non-invasive ventilation; prophylactic and therapeutic anticoagulation; and procalcitonin and antibiotic therapy. The section on transfusion therapy addressed indications and methods of transfusion, as well as the diagnosis and prediction of delayed hemolytic transfusion reactions. A total of 45 recommendations were proposed, including 14 specific to adults, 13 specific to pediatrics, and 18 applicable to both adults and children, along with three therapeutic algorithms. The Grade of Recommendation Assessment, Development, and Evaluation (GRADE) methodology was adhered to throughout the process. Sixteen recommendations were based on a low level of evidence (GRADE 2+ or 2−), while 26 were based on evidence that could not be classified under the GRADE system and were therefore considered expert opinions. Finally, for three aspects of sickle cell disease management, the experts concluded that no reliable recommendations could be made based on the current state of knowledge. The recommendations and therapeutic algorithms received strong agreement from the experts.

**Supplementary Information:**

The online version contains supplementary material available at 10.1186/s13613-025-01479-3.

## Introduction

Sickle cell disease (SCD), one of the most common monogenic disorders worldwide, is caused by a mutation in hemoglobin. When deoxygenated, sickle hemoglobin tends to polymerize within red blood cells, leading to their deformation and rigidity (sickling), which can cause vascular obstruction and result in the typical vaso-occlusive pain crisis (VOC). VOC is the most frequent acute complication of SCD, while acute chest syndrome (ACS), which can develop from VOC, is the leading cause of death in adults with SCD [1]. In addition to VOC and ACS, various other acute events can necessitate the admission of SCD patients to emergency departments or intensive care units (ICUs) [2].

Published between 2010 and 2015, the current French recommendations for managing SCD have not been formally validated by professional scientific societies. While the available literature on the management of acute complications of SCD is limited, this area has been the focus of recent, dynamic research. The French Intensive Care Society (Société de Réanimation de Langue Française, SRLF), the French Group of Pediatric Intensive Care and Emergency Medicine (Groupe Francophone de Réanimation et d’Urgences Pédiatrique, GFRUP), and the French Society for Emergency Medicine (Société Française de Médecine d’Urgence, SFMU) have organized a formal guideline process for managing emergencies and critical illnesses in patients with SCD.

The purpose of these guidelines, based on an analysis of the evidence in the literature, is to outline diagnostic strategies, patient referrals, and therapeutic management in emergency and intensive care settings.

## Methods

These recommendations are the result of the collaborative efforts of an expert panel convened by the SRLF, the GFRUP and the SFMU. The group’s agenda was predetermined, beginning with the identification by the organizing committee in consultation with coordinators of 22 key questions. Subsequently, experts were assigned to address each question. The questions were framed using the PICO format (Patient Intervention Comparison Outcome) following an initial expert group meeting.

A level of evidence was defined for each publication cited as a function of the study design. It could be revised by taking into account the methodological quality of the study. A global level of evidence was determined for each endpoint by taking into account the levels of evidence of each publication, the consistency of the results between the various studies, the direct or indirect nature of the evidence, and the cost analysis.

A"strong"overall level of evidence led to the formulation of a"strong"recommendation (must do, must not do … GRADE 1+ or 1−). An overall level of evidence categorized as"moderate,""low,"or"very low"resulted in an"optional"recommendation (probably should do, probably should not do, … GRADE 2+ or 2−). In cases where the literature was absent or insufficient, the question could be addressed with an expert opinion (experts propose …).

Proposed recommendations were presented and discussed one by one. The purpose of this process was not necessarily to inevitably reach a unique, convergent expert consensus on all of the proposals, but rather to define points of concordance, divergence or indecision. Each recommendation was then evaluated by each of the experts, who provided an individual score using a scale ranging from 1 (complete disagreement) to 9 (complete agreement). The collective score was established according to a GRADE grid methodology. To obtain strong agreement, 70% of the experts had to agree with the recommendation. In the absence of a strong consensus, the recommendations were reformulated and rescored in order to reach a consensus. Only the expert opinions that obtained a strong agreement were finally adopted.

Five fields of recommendations were defined: (1) Referral of patients; (2) VOC; (3) ACS; (4) Transfusion therapy; (5) Priapism. A literature search was conducted using the MEDLINE via the PubMed and Cochrane databases. Publications were included in the analysis if they were in English or French. The analysis focused on recent data in order of preference, from meta-analyses and randomized trials to observational studies.

## Results

The process resulted in the development of 45 guidelines, including including 14 specific to adults, 13 specific to pediatrics, and 18 applicable to both adults and children, along with three therapeutic algorithms. For three PICOs, the experts determined that no reliable recommendations could be made given the current state of knowledge. After two rounds of scoring, strong agreement was reached for all guidelines. Twenty-six guidelines were expert opinions. Table 1 provides a summary of these recommendations.

**Table 1:** Summary of recommendations

Recommendation		Level of proof
Admission to ICU		
R1.1	*Adults:* Admission to ICU should probably be considered in adults with SCD who need red blood cell exchange or are at risk of an unfavourable outcome, such as those with tachypnea, acute kidney injury, or hypotension	Grade 2+, Strong agreement
R1.2	*Paediatrics:* The experts suggest considering ICU admission for children with SCD in cases of uncontrolled hyperalgic crisis, poorly tolerated acute anemia, acute neurological event, hypoxemic respiratory distress, or any acute organ failure	Expert opinion, Strong agreement
Reference centers		
R2	The experts recommend consulting a reference center for an expert opinion or arranging a transfer for adult and paediatric patients with severe acute complications of SCD or those at risk of rapid deterioration While the list of these complications is not exhaustive, it includes: - Any acute organ failure requiring admission to the ICU or HDU; - ACS, especially if requiring respiratory support (high-flow oxygen, CPAP, non-invasive or invasive ventilation) or if associated with pulmonary hypertension (tricuspid regurgitant jet velocity > 3 m/s or acute cor pulmonale); - Acute anemia with a high risk, suspicion or confirmation of delayed haemolytic transfusion reaction (DHTR); - Stroke (hemorrhagic or ischemic); - Severe hepatic VOC, sequestration, or cholestasis (with encephalopathy, prothrombin time < 50%, or total bilirubin > 15 mg/dL or 250 micromol/L); - Extensive medullary necrosis, suggested by severe VOC, potentially with neurological signs, a significant decrease in haemoglobin and platelets (typically < 100 G/L), and a marked increase in lacticodeshydrogenase (typically > 2000 IU/L); - Splenic sequestration (usually suggested by a painful increase in spleen size and a decrease of more than 2 g/dL in haemoglobin concentration); - Acute complication in a transplanted patient (kidney, liver, heart); - Acute complication in a pregnant woman; - Sepsis or septic shock; meningitis in children; - Acute priapism; - Acute complication requiring surgery; - Sudden deafness or blindness	Expert opinion, Strong agreement
Vaso-occlusive crisis		
Pain relief		
R3.1	*Adults:* The experts suggest immediately initiating a protocolized analgesic treatment algorithm (Fig. 1) in adults with SCD presenting with a painful crisis	Expert opinion, Strong agreement
R3.2	*Paediatrics:* A protocolized analgesic treatment algorithm (Fig. 2) should probably be initiated immediately in children with SCD presenting with a painful crisis	Grade 2+, Strong agreement
Oxygen saturation target		
R4	The experts suggest maintaining a transcutaneous oxygen saturation target of 95% during VOC in adults and children with SCD	Expert opinion, Strong agreement
Incentive spirometry		
R5	Incentive spirometry should probably be used to prevent the onset of ACS in adults and children with SCD who experience VOC, especially in cases of chest or back pain in adults	Grade 2+, Strong agreement
Hydration		
R6.1	Systematic overhydration is probably not advisable in adults and children with SCD experiencing VOC, due to the potential for serious complications and the lack of demonstrated benefits	Grade 2-, Strong agreement
R6.2	*Adults:* The experts suggest adjusting fluid intake, whether oral or intravenous in adults with VOC, based on the patient's clinical status (hydration, respiratory), other fluid inputs (such as erythrocyte transfusions), and daily fluid requirements	Expert Opinion, Strong agreement
R6.3	*Paediatrics:* The experts suggest initially limiting fluid intake in children with VOC to 1.5L/m^2^/day, without exceeding 2L/m^2^/day (or a maximum of 3L/day)	Expert Opinion, Strong Agreement
Acute chest syndrome		
Lung ultrasound		
R7.1	*Adults:* Lung ultrasound should probably be used to improve the diagnosis of ACS in adults with SCD exhibiting clinical signs suggestive of ACS	Grade 2+, Strong Agreement
R7.2	*Paediatrics:* Either lung ultrasound or standard chest X-ray should probably be used for the diagnosis of ACS in children with SCD exhibiting clinical signs suggestive of ACS	Grade 2+, Strong Agreement
CT scan		
R8.1	*Adults*: The experts suggest that, after assessment of pretest clinical probability, adults with ACS undergo a CT scan to search pulmonary artery thrombosis	Expert opinion, Strong Agreement
R8.2	*Pediatrics*: The experts suggest that children admitted to the PICU with severe ACS complicated by right ventricular failure undergo a CT scan to search pulmonary artery thrombosis	Expert opinion, Strong Agreement
Echocardiography		
R9.1	*Adults*: Right ventricular stress should probably be assessed with echocardiography for prognostic evaluation in adult patients with severe ACS	Grade 2+, Strong Agreement
R9.2	*Paediatrics:* The experts suggest that in children, right ventricular stress be assessed with echocardiography for prognostic evaluation in pediatric patients with severe ACS	Expert opinion, Strong Agreement
Systemic corticosteroids		
R10	Systemic corticosteroid therapy should probably not be used routinely for the management of ACS in adults and children with SCD	Grade 2-, Strong agreement
Non-invasive ventilation		
R11.1	*Adults*: Non-invasive ventilation should probably not be used routinely during ACS in adults with SCD	Grade 2-, Strong agreement
R11.2	*Paediatrics*: The experts suggest testing the efficacy and tolerance of non-invasive ventilation on a case-by-case basis during ACS in children with SCD	Expert opinion, Strong agreement
Inhaled nitric oxide		
R12	High-dose nitric oxide should probably not be used routinely for the management of adults and children with SCD developing VOC or ACS	Grade 2-, Strong Agreement
Therapeutic anticoagulation		
R13	Currently available data do not allow a recommendation to be made regarding the use of therapeutic anticoagulation in adult and pediatric patients with ACS without documented deep vein thrombosis or pulmonary artery thrombosis	No specific recommendation
Prophylactic anticoagulation		
R14.1	*Adults*: Thromboprophylaxis is indicated in acutely ill medical patients, including those with ACS	No specific recommendation
R14.2	*Paediatrics*: Prophylactic anticoagulation should probably be used in children with SCD with ACS in the following cases: pubertal onset, a history of deep vein thrombosis, central venous catheterization, COVID-19, or Moya-Moya disease (after an individual assessment of the risk–benefit ratio)	Grade 2+, Strong Agreement
Procalcitonin		
R15	The panel makes no recommendation on procalcitonin for initiating antibiotic treatment in adult and children with ACS	No recommendation, Strong agreement
Antibiotics for ACS		
R16.1	*Adults*: The experts suggest administering empirical antibiotic targeting intracellular bacteria in combination with an antibiotic targeting pyogenic bacteria in cases of severe ACS in adults	Expert opinion, Strong agreement
R16.2	*Paediatrics*: Empirical antibiotic therapy targeting intracellular pathogens should probably be used in combination with an antibiotic targeting pyogenic bacteria in children with ACS	Grade 2+, Strong agreement
Intravenous beta-lactams in patients with SCD		
R17	In adult and pediatric patients with SCD experiencing severe or difficult-to-treat infections, and without renal insufficiency, intravenous beta-lactams should probably be administered using a loading dose followed by continuous administration, or an increased number of doses per day, in order to improve their pharmacokinetics and pharmacodynamics	Grade 2+, Strong agreement
Transfusion therapy		
Transfusion modality		
R18.1	In the event of an acute complication warranting a transfusion in an adult or paediatric patient with SCD, the experts suggest prioritizing simple transfusion if the anemia is severe (total hemoglobin < 7 g/dL) and immediately performing an exchange transfusion (manual or automated) in other cases	Expert opinion, Strong agreement
R18.2	*Paediatrics:* In the case of an acute complication warranting a transfusion in a child with SCD, the experts suggest not to exceed a post-procedure hematocrit of 33% ± 3%	Expert opinion, Strong agreement
Indications for blood transfusion		
R19.1	The experts suggest against the systematic use of transfusion therapy for uncomplicated VOC in adult and paediatric patients with SCD	Expert opinion, strong agreement
R19.2	*Adults*: The experts suggest performing a transfusion therapy for the management of ACS in adult patients with SCD who meet severity criteria with regard to hypoxemia, severe pulmonary hypertension (tricuspid regurgitation velocity > 3 m/s or acute cor pulmonale), or any associated organ failure	Expert opinion, Strong agreement
R19.3	*Pediatrics*: The experts suggest performing a transfusion therapy for the management of ACS in pediatric patients with SCD who meet severity criteria, with regard to rapidly worsening hypoxemia, or any associated organ failure	Expert opinion, Strong agreement
R19.4	The experts suggest urgent transfusion therapy, ideally within two hours of symptom onset, for adult and paediatric patients with SCD showing signs of stroke. Automated exchange transfusion should be prioritized if time allows, or manual exchange if there are delays in accessing automated techniques. In cases where haemoglobin levels are below 7 g/dl, a simple transfusion is suggested while awaiting exchange transfusion to reach haemoglobin S (or S + C) below 30% with haematocrit 30 ± 3% Haemorrhagic stroke may also require exchange transfusion or simple transfusion, especially when neurosurgical procedures are indicated	Expert opinion, Strong agreement
R19.5	The experts suggest transfusion therapy for persistent acute ischemic priapism in adult and paediatric patients with SCD when non-invasive and invasive treatments (aspiration and/or intracavernosal injection) fail or when priapism has been ongoing for at least four hours	Expert opinion, Strong agreement
R19.6	The experts suggest transfusion therapy in cases of splenic sequestration in adult and paediatric patients with SCD	Expert opinion, Strong agreement
R19.7	The experts suggest transfusion therapy in the event of acute organ failure	Expert opinion, Strong agreement
R19.8	Transfusion therapy should probably not be performed in adult and pediatric patients with SCD presenting with Delayed Haemolytic Transfusion Reaction	Grade 2-, Strong Agreement
Delayed Haemolytic Transfusion Reaction		
Diagnosis		
R20.1	*Adults*: The Mekontso Dessap nomogram should probably be used to diagnose DHTR in adult patients with SCD	Grade 2+, Strong agreement
R20.2	*Paediatrics*: The experts suggest using the Mekontso Dessap nomogram to diagnose DHTR in children with SCD	Expert opinion, Strong agreement
R20.3	The experts suggest systematically performing a complete blood count and haemoglobin electrophoresis within 48 h following RBC transfusion in adult and pediatric patients with SCD at risk of DHTR	Expert opinion, Strong agreement
Prediction		
R21.1	*Adults*: The experts suggest using the Narbey predictive score to assess the risk of DHTR before transfusion in adult patients with SCD. They suggest categorizing the risk as: i) not significant if the score is less than 8; and ii) significant if the score equals or exceeds 8, or if the patient has a history of confirmed DHTR	Expert opinion, Strong agreement
R21.2	*Paediatrics*: The Narbey predictive score should probably not be used in children with SCD to assess the risk of DHTR	Grade 2-, Strong agreement
Priapism		
R22.1	*Adults*: In cases of persistent, painful erection in an adult with SCD suggesting acute ischemic priapism, experts recommend promptly initiating non-invasive measures (such as massages, physical exertion, and possibly oral alpha-adrenergic agonists). If priapism lasts for more than an hour, an intracavernosal injection of alpha-adrenergic agonists should be considered, with a repeat injection after 20 min if the first is ineffective, the objective being to minimize complications and long-term sequelae	Expert opinion, Strong agreement
R22.2	*Adults*: For ischemic priapism persisting for four hours or more in adults with SCD, experts recommend aspirating cavernosal blood, followed by an intracavernous injection of alpha-adrenergic agonists. These steps should be repeated multiple times before considering surgical intervention, the objective being to minimize complications and long-term sequelae	Expert opinion, Strong agreement
R22.3	*Paediatrics:* Experts suggest performing an intracavernous injection of alpha-adrenergic agonists and draining the corpora cavernosa in a child with SCD who has been experiencing acute priapism for more than an hour	Expert opinion, Strong agreement

### First area: referral of patients

R1. Admission to ICU

*R1.1: Adults*: Admission to ICU should probably be considered in adults with SCD who need red blood cell exchange or are at risk of an unfavourable outcome, such as those with tachypnea, acute kidney injury, or arterial hypotension. (Grade 2+, strong agreement).

*R1.2: Pediatrics*: The experts suggest considering ICU admission for children with SCD in cases of uncontrolled hyperalgic crisis, poorly tolerated acute anemia, acute neurological event, hypoxemic respiratory distress, or any acute organ failure. (Expert opinion, strong agreement).

*Rationale*

ICU admission should generally be considered when there are clinical or biological signs of severe illness, which are common to any critical condition and not specific to SCD. Patients with SCD are at risk for severe complications, such as ACS, stroke, and splenic or hepatic sequestration, which can lead to significant morbidity and may require ICU or high-dependency unit (HDU) care. Many of these complications often necessitate red blood cell (RBC) exchange.

*Adults*: Two retrospective studies have examined the risk of adverse outcomes (death or need for organ support) in adult SCD patients admitted to the ICU. Cecchini et al. found that patients with poor outcomes had more tachypnea, acute renal failure, and decreased haemoglobin levels within 48 h before ICU admission [3]. Agbakou et al. identified tachypnea, acute renal failure, arterial hypotension, higher haemoglobin level, and RBC exchange before ICU transfer as predictors of adverse outcomes [4]. In general, ICU admission should be considered in cases of any organ failure, including neurological disorders.

*Paediatrics*: Five retrospective studies have described the stays of children with SCD in Pediatric Intensive Care Unit (PICU) [5–8]. Clinical experience from high-volume centers suggests PICU admission for uncontrolled hyperalgic crises, poorly tolerated acute anemia, acute neurological events, hypoxemic respiratory distress, or any acute organ failure [9]. These centers emphasize vigilance in cases of rapidly progressing organ failure (respiratory, hemodynamic, neurological) or severe acute anemia, in order to enhance monitoring or provide organ support.

R2: Reference centers

The experts recommend consulting a reference center for an expert opinion or arranging a transfer for adult and pediatric patients with severe acute complications of SCD or those at risk of rapid deterioration (Expert opinion, strong agreement).

While the list of these complications is not exhaustive, it includes:

- Any acute organ failure requiring admission to the ICU or HDU;
- ACS, especially if requiring respiratory support (high-flow oxygen, continuous positive airway pressure, non-invasive or invasive ventilation) or if associated with pulmonary hypertension (tricuspid regurgitant jet velocity > 3 m/s or acute cor pulmonale);
- Acute anemia with a high risk, suspicion or confirmation of delayed haemolytic transfusion reaction (DHTR);
- Stroke (hemorrhagic or ischemic);
- Severe hepatic VOC, sequestration, or cholestasis (with encephalopathy, prothrombin time < 50%, or total bilirubin > 15 mg/dL or 250 micromol/L);
- Extensive medullary necrosis, suggested by severe VOC, potentially with neurological signs, a significant decrease in haemoglobin and platelets (typically < 100 G/L), and a marked increase in lacticodeshydrogenase (typically > 2000 IU/L);
- Splenic sequestration (usually suggested by a painful increase in spleen size and a decrease of more than 2 g/dL in haemoglobin concentration);
- Acute complication in a transplanted patient (kidney, liver, heart);
- Acute complication in a pregnant woman;
- Sepsis or septic shock; meningitis in children;
- Acute priapism;
- Acute complication requiring surgery;
- Sudden deafness or blindness.

*Rationale*

Seeking care from an expert center for severe complications in patients with SCD can improve diagnosis and treatment and enable transfer in cases of rapid deterioration. Patients with SCD can develop acute complications that may worsen quickly without proper management. The most serious complications reported include acute organ failures, ACS, acute anemia, stroke, hepatic VOC, sequestration, or cholestasis, medullary necrosis, splenic sequestration, sepsis, acute priapism, and sudden deafness or blindness. Patients with SCD are particularly vulnerable in cases of transplantation, pregnancy, and peri-operative conditions. Early diagnosis and management are crucial in view of reducing morbidity and mortality. Due to the rarity and complexity of SCD, expert teams are particularly prone to rapidly identify, assess and manage some very rare complications. Some interventions, such as specific transfusions (e.g., extended phenotyping) or medications (e.g., immunomodulation for DHTR), may not be available in all hospitals.

*Adults*: Although no RCTs exist on this topic, it is reasonable to refer adult patients with severe complications of SCD to a specialized center. This could involve expert consultation or patient transfer, depending on the type of complication, clinical condition, distance, and capacity of the referral center.

*Paediatrics*: Raphael et al. conducted a study comparing the length of stay for 35 paediatric admissions in a day hospital (DH) versus conventional hospitalization for uncomplicated VOC, and found a 39% reduction in length of stay in the DH group [10]. Karkoska et al. found better adherence to guidelines and fewer admissions when managing VOC in a paediatric haematology DH versus the emergency department [11]. Another study by Raphael et al. analyzed PICU transfer rates for 83,477 hospitalizations of children with SCD, and showed lower transfer rates and shorter length of stay in high-activity SCD centers, regardless of overall activity level [12]. Jan et al. compared the ACS management in general hospitals versus paediatric hospitals using data from 1476 adolescents and young adults [13]. They found longer length of stay and higher intubation rates in general hospitals, which may be attributed to the specialized care in paediatric hospitals and/or the older age of patients treated in general hospitals. Despite a low level of evidence, North American guidelines recommend consulting an expert center for severe complications in children with SCD [14].

### Second area: VOC

R3: Pain relief

**Fig. 1:**
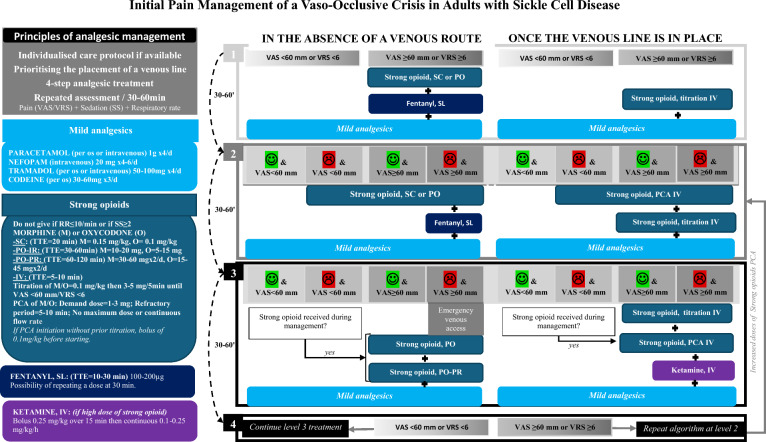
Initial pain management of a vaso-occlusive crisis in adults with sickle cell disease. *PCA *patient controlled analgesia, *VAS *visual analog scale, *VRS *verbal rating scale, *RR *respiratory rate; *SS *sedation scale, *1 *drowsy patient, easily awakened, *2 *very drowsy, awakened by verbal stimulation, *3 *very drowsy, awakened by tactile stimulation, *SL *sublingual, *SC *subcutaneous, *PO *per os, *IV *intravenous, *IR *immediate release, *PR *prolonged release, *TTE *time to effect, *green smiley* improvement, *red smiley* no improvement

R3.1: *Adults:* The experts suggest immediately initiating a protocolized analgesic treatment algorithm (Fig. 1) in adults with SCD presenting with a painful crisis. (Expert opinion, strong agreement).

*Analgesics for adult patients with SCD*

A summary of analgesic treatments for pain relief in adult patients with SCD in an emergency and critical care setting is presented in Table 2.

**Table 2:** Analgesic treatments for adult patients with SCD in the emergency and critical care setting

Class	Medications	Remarks	Recommendation	Grade	Agreement
Anti-nociceptive					
Non-opioids	Paracetamol	Check total consumption at home	Recommended	Expert opinion	Strong
	NSAIDs		Not recommended as first-line. Experts suggest using NSAIDs on an individualized basis, especially considering suspected triggers and the patient's history (good usual therapeutic response, renal insufficiency, etc.)	GRADE 2-	Strong
Weak opioids*	Codeine	Do not use if a strong opioid is needed	Recommended	Expert opinion	Strong
	Tramadol	Can be combined with a strong opioid	Recommended	Expert opinion	Strong
Strong opioids**	Morphine Oxycodone	Do not wait for obtaining a vein to start treatment	Recommended immediately if severe pain (VAS ≥ 60 mm) or refractory to strong non-opioid analgesics Experts suggest prioritizing the administration of strong opioids intravenously using the PCA method early	Expert opinion	Strong
	Fentanyl (sublingual)	Limit to 1 or 2 maximum doses (short duration of action)	Recommended for severe pain (VAS ≥ 60 mm) without immediate vein access	Expert opinion	Strong
	Buprenorphine	To be continued in long-term treated patients	Not recommended	Expert opinion	Strong
Anti-hyperalgesic	Nefopam		Recommended	Expert opinion	Strong
	Ketamine	Requires close monitoring Always in combination with morphine	Recommended when high doses of strong opioids are needed (≥1 mg/kg oral morphine equivalent over a 4-h period) Experts suggest the following usage modality: a slow 0.25 mg/kg IV bolus followed by a continuous infusion between 0.1 and 0.25 mg/kg/h	Expert opinion	Strong
	Nitrous oxide	Maximum 1 h cumulative per day	Not recommended as a first-line treatment Reserved for painful procedures	Expert opinion	Strong
Modulator of transmission and peripheral sensitization	Local lidocaine		Recommended for neuropathic pain elements and localized pain	Expert opinion	Strong

*NSAID* non-steroidal anti-inflammatory drug, *VAS* visual analog scale

*Avoid if there is suspicion of ultra-rapid metabolizer (risk of toxicity) or slow metabolizer (risk of inefficacy); avoid combining weak opioids with each other

**Morphine or oxycodone depending on the unit's protocols; do not administer strong opioids if the sedation scale ≥ 2 or respiratory rate ≤ 10/min

*Rationale*

SCD is often marked by frequent painful VOCs, making effective pain management critical. Experts outline five core principles known as the"5 P’s": early (Precocious) initiation of pain treatment, Plurality of strong pain-relieving agents, Personalized treatment plans, Participatory decision-making, and Psychological considerations.

*Precocious initiation of pain treatments*: Prompt pain treatment is essential to alleviate pain and prevent complications such as ACS. Studies suggest that early and maximal pain management, including pain titration and regular reassessments, helps break the pain cycle in VOC [15–18]. Alternative administration routes (sublingual, oral, and subcutaneous) are useful while attempting to establish venous access [15, 18]. Intravenous administration of strong opioids is often necessary, but studies on different intravenous methods (continuous, patient-controlled analgesia [PCA], on-demand) show varied results [19–23]. PCA is a safe method that may reduce morphine consumption and side effects and lessen the workload of nursing staff by reducing the need for rescue doses in emergency settings [22].

*Plurality of strong pain-relieving agents*: To minimize opioid side effects and address the complex pain mechanisms in VOC, combining non-opioid analgesics like paracetamol, nefopam, and tramadol with strong opioids is advised. While not specifically evaluated for VOC, these drugs have demonstrated morphine-sparing effects in non-sickle cell patients [24]. Three RCTs on non-steroidal anti-inflammatory drugs (NSAIDs) as co-analgesics with morphine in VOC showed mixed results [25, 26], with the most recent study [27] finding no reduction in morphine use or pain intensity. Despite mixed evidence, recent guidelines [28] and reviews [29] suggest short-term NSAID use alongside strong opioids, with careful consideration of the risk of nephrotoxicity. A review of nearly 500 patients treated for VOC (mostly case series) suggests that when used with morphine, low-dose ketamine, is effective for severe pain with acceptable tolerability, particularly when administered intermittently [30]. To minimize adverse effects, especially psychodysleptic reactions, a slow initial bolus (over 15 min) is recommended [31].

*Personalized and participatory approach*: Pain treatment should be tailored to individual responses and genetic factors. Medications like codeine and tramadol, metabolized by CYP2D6, show variable efficacy due to genetic polymorphisms [32]. Evaluating patient response through anamnesis can guide whether to include these drugs -which are commonly used in home settings [33]- in the treatment plan. A personalized approach and shared decision-making between patients and healthcare providers can enhance treatment outcomes [34]. A RCT demonstrated that personalized care protocols improved pain scores and reduced hospital stays [17]. Continual assessment of pain mechanisms and analgesic strategies, considering factors such as opioid-induced hyperalgesia and tolerance, is crucial.

*Psychological considerations*

Effective communication is vital to ensure quality care and psychological well-being [35]. Misunderstandings about opioid use, such as assumptions about abuse or disbelief in pain severity, can impede pain management [36–39]. Terms like dependence, abuse, and tolerance are often confused, leading to risks of undertreatment [38]. In France, less than 10% of patients use strong opioids at home [33], and the high opioid doses needed for pain relief are frequently due to increased morphine clearance rather than tolerance or abuse [40].

Overall, a holistic approach to managing pain during VOCs in SCD is essential, considering individual differences, psychological factors, and the appropriate use of analgesics.

**Fig. 2:**
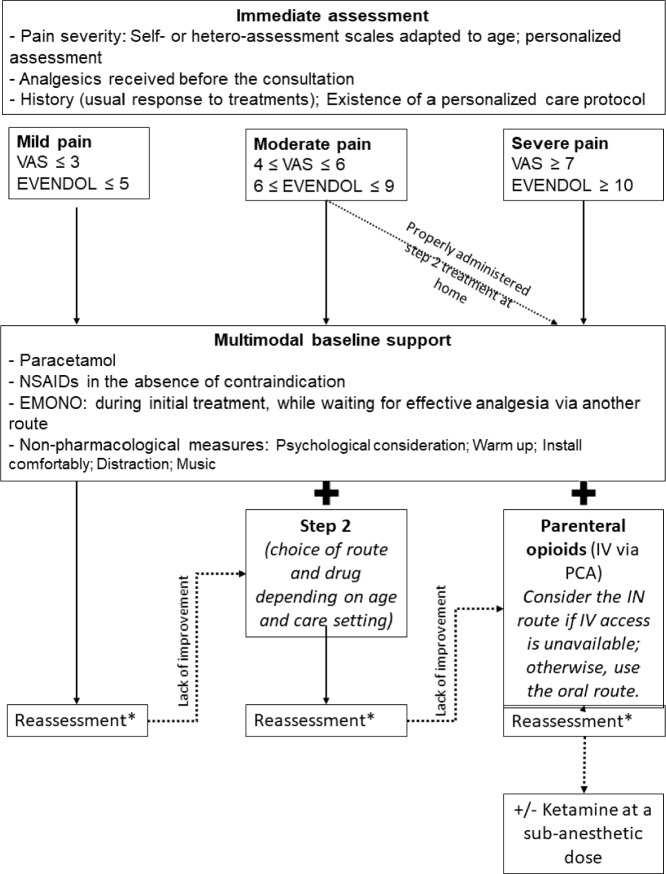
Initial pain management of a vaso-occlusive crisis in children with sickle cell disease. *VAS *visual analog scale, *EMONO *equimolar mixture of oxygen and nitrous oxide, *NSAID *non steroidal anti-inflammatory, *EVENDOL *évaluation enfant douleur, *PCA *patient controlled analgesia, *IV *intravenous, *IN *intranasal, *PO *per os,*every 30–60 min (personalized and based on age)

R3.2: Paediatrics: A protocolized analgesic treatment algorithm (Fig. 2) should probably be initiated immediately in children with SCD presenting with a painful crisis. (Grade 2+, strong agreement).

*Analgesics for paediatric patients with SCD*

A summary of analgesic treatments for pain relief in paediatric patients with SCD in the emergency and critical care setting is presented in Table 3.

**Table 3:** Analgesic treatments for pediatric patients with SCD in the emergency and critical care setting

Molecule/method of delivery	Recommandation	Grade	Agreement
Tier 1			
Acetaminophen	The experts suggest routine use of paracetamol in children with SCD experiencing a VOC	Expert opinion	Strong
Non-steroidal anti-inflammatory drugs (NSAIDs)	The experts suggest short-term use of NSAIDs in children with SCD experiencing a VOC, provided there is no dehydration, abdominal pain, or contraindication for NSAIDs	Expert opinion	Strong
Tier 2			
Tramadol, Codeine, Nalbuphine hydrochloride	The experts suggest using step 2 analgesics (Tramadol, Codeine, Nalbuphine hydrochloride) if paracetamol fails to relieve mild to moderate pain in children with SCD experiencing a VOC	Expert opinion	Strong
Codeine	The experts, in agreement with the ANSM, suggest no longer using this molecule in children under 12 years old due to the risk of serious adverse events	Expert opinion	Strong
Morphinics			
Opioids	The experts suggest promptly using opioid analgesics for any severe pain, as well as for pain that is resistant to step 1 and step 2 analgesics, in children with SCD experiencing a VOC	Expert opinion	Strong
Intravenous (IV): Morphine, Oxycodone	It is likely necessary to use the intravenous route as soon as a vein is accessible and to favour a prescription that combines a basal infusion rate with bolus doses using Patient-Controlled Analgesia	Grade 2+	Strong
Other routes:—intranasal: Fentanyl, Sufentanil- oral: Morphine	The experts suggest using routes other than the IV route (such as intranasal or oral) during initial hospital management, as this can facilitate the rapid administration of opioids in children with SCD experiencing a VOC	Expert opinion	Strong
Other molecules			
Ketamine at sub-anesthetic dose	Ketamine should likely be used in hospitalized children with SCD experiencing a VOC, especially when they have refractory pain or pain that is inadequately relieved by a combination of acetaminophen and opioids	Grade 2+	Strong
Equimolar Mixture of Oxygen and Nitrous Oxide	Experts recommend short-term use of an equimolar mixture of oxygen and nitrous oxide during the initial phase of hospital management for children with SCD experiencing a VOC, while waiting for effective pain relief from another route of administration	Expert opinion	Strong
	Experts do not provide any recommendations regarding use or non-use of an equimolar mixture of oxygen and nitrous oxide subsequent to the initial phase of hospital management in children with SCD experiencing a VOC	Lack of recommendation	–
Inhaled nitric oxide	Experts do not provide any recommendation regarding use or non-use of inhaled nitric oxide in children with SCD experiencing VOC	Lack of recommendation	–
Refractory pain	Experts suggest using alternative non-opioid or opioid-sparing analgesics, particularly Nefopam (despite the lack of marketing authorization), in children with SCD experiencing a VOC	Expert opinion	Strong
Non-pharmacological measures			
Music, Relaxation, Massages, Yoga, Virtual reality…	Experts suggest combining non-pharmacological measures, such as music therapy, relaxation techniques, and virtual reality, in children with SCD experiencing a VOC, if these methods are known and accepted by the patient and the healthcare team	Expert opinion	Strong

*SCD* sickle cell disease, *VOC* vaso-occlusive crisis, *NSAID* non-steroidal anti-inflammatory drug, *ANSM* Agence Nationale de Sécurité du Médicament

*Rationale*

The systematic literature review identified 20 RCTs evaluating immediate analgesic treatment for VOC in children (0–18 years) and analyzed 25 observational studies for these recommendations. Due to the heterogeneity and low evidence level of these studies, the recommendations complementarily rely on pharmacological knowledge from other pediatric cases and experience drawn from observational studies and expert centres.

*Paracetamol and NSAIDs*: Three studies, including two RCTs, assessed paracetamol for VOC in children. A placebo-controlled RCT showed no analgesic benefit or reduction in opioid use with acetaminophen when combined with intravenous morphine and/or ketorolac [41]. Despite this, due to its low toxicity, paracetamol is suggested as a first-line treatment in combination with other analgesics. Three studies have examined NSAIDs: while one RCT found no benefit of ketorolac over placebo for pain relief or reduced opioid use when combined with morphine [42], another RCT found piroxicam to be superior to aspirin for pain reduction [43]. A prospective observational study reported pain relief in 53% of cases after ketorolac administration, without a need for opioids [44].

*Tramadol, codeine, nalbuphine hydrochloride*: Expert recommendations on these drugs are based on findings in other contexts since only one retrospective study has shown similar effectiveness using nalbuphine and meperidine in children with SCD [45].

*Opioids*: Two observational studies suggest that early opioid use reduces time to effective analgesia [46, 47]. The optimal initial administration of intravenous morphine using PCA remains unclear due to inconsistent findings on different dosing strategies [48–50]. However, high bolus doses with a lower basal rate may reduce total morphine requirements [49, 50]. Early opioid administration, ideally within 30 min, is crucial insofar as delays worsen outcomes, including pain scores, cumulative dose of opioids, and length of stay [51] [52]. Intranasal fentanyl was found in a RCT to be superior to placebo for 20-min pain relief [53]. Observational studies suggest that intranasal opioid administration reduces time to first dose [54–56]. An RCT comparing oral and intravenous morphine is difficult to interpret due to differences in intravenous loading doses [57]. While observational studies have identified a negative association between oral morphine use and hospitalization rates [58, 59], methodological limitations restrict the strength of these conclusions.

*Ketamine, equimolar mixture of oxygen and nitrous oxide, inhaled nitric oxide and nefopam*: One RCT found no significant pain reduction benefit with ketamine at sub-anesthetic dose compared to intravenous morphine [60], while other observational studies reported decreased pain scores and/or opioid needs with ketamine [61–64]. Limited evidence favours an equimolar mixture of oxygen and nitrous oxide in children with SCD. While a small RCT showed a benefit of inhaled nitric oxide on pain after four hours [65], a Cochrane review found insufficient evidence justifying its use in VOC [66]. Recommendations on nefopam are based on extrapolation from other contexts, as there are no pediatric data for SCD, and it has not been authorised for use in children.

*Non-pharmacological measures and other interventions*: Limited data exist on non-pharmacological measures (e.g., music, relaxation, massages, yoga, virtual reality) for children with SCD, but evidence from other populations suggests they may be beneficial if accepted by the patient and the team [67–70]. Transcutaneous electrical nerve stimulation (TENS, one RCT) [71] and magnesium (2 RCTs) [72, 73] are lacking in demonstrated benefits for VOC. One RCT showed a significant reduction in the length of stay with dexamethasone, but an increased risk of rehospitalization [74].

*Personalised approach and psychological considerations*: As in adults, pain management should be tailored to each patient, with individualized care protocols potentially reducing pain and opioid duration [75]. Psychological support, including family presence, is crucial for managing VOC in children, with adaptations based on age.

R4: Oxygen saturation target

The experts suggest maintaining a transcutaneous oxygen saturation target of 95% during VOC in adults and children with SCD. (*Expert opinion, strong agreement*)

*Rationale*

Several arguments support oxygen therapy during VOC. In vitro studies have shown that oxygen desaturation worsens red blood cell sickling. Since continuous monitoring of oxygen saturation is often lacking in hospital wards, routine supplemental oxygen may help to prevent desaturation, particularly at night. One study found an association between nighttime desaturation in a steady state and an increased annual number of painful crises in children [76].

However, there are also arguments against the routine use of supplemental oxygen during VOC. It may mask early detection of desaturation, which could be a warning sign for ACS. Additionally, hyperoxia could be harmful due to the oxidation of red blood cells. Oxygen therapy can limit patient mobility, cause discomfort, and complicate outpatient crisis management.

To date, only three studies with methodological limitations have evaluated the effect of routine oxygen therapy versus room air in treating VOC in adults and children with SCD [77–79]. These studies found no differences in hospitalization duration or opioid use. However, the level of evidence was very low due to several limitations, including single-center settings, open-label designs, and small sample sizes in two of the studies [77, 79]. Additionally, discrepancies in transcutaneous oxygen saturation measurements by different methods have been reported in patients during VOC [80]. Various guidelines recommend supplemental oxygen therapy to manage VOC, but thresholds for initiating oxygen differ: less than 95% in the USA [14] and French paediatric guidelines [81], or less than 96% in the UK [82] and French adult [83] guidelines. We suggest initiating oxygen therapy when pulse oximetry drops below 95% on room air in adults and children with VOC.

R5: Incentive spirometry

Incentive spirometry should probably be used to prevent the onset of ACS in adults and children with SCD who experience VOC, especially in cases of chest or back pain in adults (*Grade 2+, strong agreement*).

*Rationale*

VOC can impair breathing in patients with SCD by reducing thoracic expansion due to pain, leading to hypoventilation and hypoxia in partially or fully collapsed lung regions. These conditions may contribute to the development of ACS [84]. Incentive spirometry, which involves regular active participation by the patient to increase tidal volume through deep inspirations throughout the day, with or without a device (such as Respiflow®, InspirX®, Coach-2®, Spiro-Ball®, etc.), may reduce the risk of progression to ACS.

*Paediatrics*: Bellet et al. conducted the only RCT comparing incentive spirometry to standard care without ventilatory techniques in children with SCD and VOC. The study involved 38 patients with SCD aged 8–21 years with dorsothoracic VOC, divided into two groups of 19 [85]. The incentive spirometry group performed 10 maximal inspirations every two hours while awake from 8 am to 10 pm, while the control group did not use incentive spirometry. The two groups were similar in age, haemoglobin levels, antibiotic use, erythrocyte transfusions, and length of stay. Incentive spirometry significantly reduced the incidence of atelectasis and pulmonary infiltrates. A paediatric cohort study before and after incentive spirometry implementation in children hospitalized for non-respiratory reasons (about 250 patients per group) showed that incentive spirometry reduced the number of transfusions and ACS in patients with dorsal VOC [86]. No adverse effects of incentive spirometry were reported. Another study found that inclusion of incentive spirometry in a care program improved outcomes by shortening hospital stays and reducing costs [87].

Recent American and British guidelines strongly recommend using incentive spirometry to prevent ACS during VOC [14, 88]. Having a physiotherapist to assist with incentive spirometry is recommended. Even though British experts believe further studies on incentive spirometry are unnecessary, a recent systematic review suggested that more research is needed. An RCT of 20 children with SCD found no difference between resistance expiration generating positive end-expiratory pressure and incentive spirometry in preventing ACS [89]. While the Bellet et al. study method remains the standard, extrinsic positive end-expiratory pressure through resistance expiration could be an effective alternative for younger, less cooperative children.

*Adults*: The benefits of incentive spirometry in adults with SCD are less clear than in children. A prospective randomized study specifically involving adults did not show a significant reduction in ACS incidence with incentive spirometry use [90]. Differences in results between paediatric and adult populations may be due to varying disease mechanisms. In practice, incentive spirometry implementation in adults is suboptimal, with only about 50% adherence [91]. Adults with back pain, a known risk factor for secondary ACS [92], might particularly benefit from incentive spirometry, but further research is needed to confirm its efficacy in this group.

R6: Hydration

R6.1: Systematic overhydration is probably not advisable in adults and children with SCD experiencing VOC, due to the potential for serious complications and the lack of demonstrated benefits (*Grade 2−, Strong agreement*).

R6.2: *Adults:* The experts suggest adjusting fluid intake, whether oral or intravenous in adults with VOC, based on the patient’s clinical status (hydration, respiratory), other fluid inputs (such as erythrocyte transfusions), and daily fluid requirements (*Expert Opinion, Strong agreement*).

R6.3: *Paediatrics*: The experts suggest initially limiting fluid intake in children with VOC to 1.5 L/m^2^/day, without exceeding 2 L/m^2^/day (or a maximum of 3 L/day) (*Expert Opinion, Strong Agreement*).

*Rationale*

The pathophysiological mechanisms of VOC involve increased blood viscosity due to the aggregation of dehydrated and sickled red blood cells [93, 94]. In vitro studies suggest that solutions with intermediate tonicity (111–122 mEq/L of sodium) allow optimal red blood cell morphology [95]. Insufficient fluid intake can exacerbate VOC by worsening dehydration. Beyond basic needs, hyperhydration aims to correct hyperviscosity in patients prone to dehydration, which results from reduced oral intake due to pain and chronic impaired urine concentration [96]. While this approach is common, no randomized study supports its effectiveness [97]. Additionally, excessive fluid intake may lead to volume overload, potentially causing cardiac dysfunction or worsening respiratory function [98]. Currently, there are no specific recommendations regarding the volume or type of maintenance fluids to administer in this context.

*Adults:* To date, no RCT has demonstrated the superiority of one hydration solution or strategy over another for VOC. A retrospective study found that hyperhydration, especially within the first 24 h, was associated with serious adverse events such as increased oxygen requirements, acute kidney injury, or ICU admissions [99]. In a Dutch registry study, where patients received three liters of hydration over 24 h, 21% experienced symptoms of fluid overload, which was associated with longer hospital stays [100].

*Paediatrics:* Only one retrospective study [101] has been identified, and it does not sufficiently address the issue. Recent international guidelines outline general principles for the use of maintenance fluids in critically ill children [102]. Despite the lack of robust scientific data to determine the ideal type and volume of maintenance fluids for children with SCD during VOC, balanced solutions with a minimum volume of 1.5 L/m^2^/day are likely preferable. Fluid intake should be adjusted based on the clinical situation and patient condition, without exceeding a total daily intake of 3 L. Further studies are needed to provide clearer guidance.

### Third area: ACS

R7: Lung ultrasound

*R7.1: Adults*: Lung ultrasound should probably be used to improve the diagnosis of ACS in adults with SCD exhibiting clinical signs suggestive of ACS. (*Grade 2+, Strong Agreement*)

*R7.2: Paediatrics*: Either lung ultrasound or standard chest X-ray should probably be used for the diagnosis of ACS in children with SCD exhibiting clinical signs suggestive of ACS. (*Grade 2+, Strong Agreement*).

*Rationale*

Today, diagnosis of ACS requires a chest X-ray (CXR) [88]. Lung ultraSound (LUS), performed by a trained operator, offers an interesting alternative to CXR (no ionizing radiation, can be conducted at the patient’s bedside in minutes). However, its use requires training and immediate access to ultrasound equipment for rapid assessment.

*Adults*: One observational study has evaluated LUS for diagnosing ACS [103] using CXR interpretation as the gold standard. LUS identified lung abnormalities sooner than CXR [103]. Another study compared the respective performances of LUS and CXR, with CT scans as the gold standard [104]. While LUS and CXR both had high specificity (89 and 95%, respectively), LUS showed much better sensitivity (72 vs. 44%) and overall agreement (0.45 vs. 0.3) with CT scans than chest X-rays. These findings were reinforced in posterior-inferior regions of the lungs.

*Paediatrics*: A study published in 2016 [105] evaluated the diagnostic performance of LUS compared to CXR in diagnosing ACS in febrile children with SCD. In this study, LUS showed sensitivity of 87% (95% CI = 62–96%), specificity of 94% (95% CI = 88–97%), a positive likelihood ratio of 14.6 (95% CI = 6.5–32.5), and a negative likelihood ratio of 0.14 (95% CI = 0.04–0.52) for diagnosing ACS. Kappa agreement coefficient in this study was 0.77. Two more recent studies likewise confirmed the good performance of LUS compared to CXR in diagnosing ACS in febrile children with SCD. Cohen et al. reported sensitivity of 88% and specificity of 93% for diagnosing ACS using LUS [106], while Preto-Zamperlini’s study [107] demonstrated overall similar performance between LUS (82%) and CXR (88%) in diagnosing ACS, but with better negative predictive value for LUS (100 vs. 77%, *p* = 0.0025). Until the definition of ACS is revised, CXR remains the current gold standard, especially in children with SCD with significant thoracic pain for whom LUS might be challenging.

R8: CT scan

*R8.1: Adults*: The experts suggest that, after assessment of pretest clinical probability, adults with ACS undergo a CT scan to search for pulmonary artery thrombosis. (*Expert opinion, Strong Agreement*).

*R8.2: Pediatrics*: The experts suggest that children admitted to the PICU with severe ACS complicated by right ventricular failure undergo a CT scan to search for pulmonary artery thrombosis. (*Expert opinion, Strong Agreement*).

*Rationale*

*Adults*: SCD is associated with hypercoagulability and frequent thrombotic events [108–112]. One observational study focused on pulmonary artery thrombosis (PAT) during ACS and found prevalence of 17% [113]. The term PAT is preferred over"pulmonary embolism"(PE) as it better reflects the primarily in situ (as opposed to embolic) nature of the thrombosis [113]. Up until now, no study has explored the prognostic impact of diagnosing and treating PAT during ACS. Therefore, experts suggest to search for PAT during ACS only after a risk assessment, and not systematically. The diagnostic strategies recommended for PE suspicion are ineffective [114]. Evaluation of PAT risk cannot rely on D-dimer levels, lower limb ultrasound Doppler, or echocardiography results. Elevated D-dimer levels are found in patients with SCD at baseline [115] and during ACS, whether or not there is PAT [113]. PAT is typically characterized by no proximal and rare (11%) distal deep venous thrombosis [113]. Pulmonary vascular dysfunction is frequently found on echocardiography during ACS, whether or not it is accompanied by PAT [113, 115]. To evaluate the risk of PAT during ACS, current data recommend not using the Geneva [116] or Wells scores [108, 117] but rather using the PAT-ACS score [116], which is based on four items: haemoglobin > 8.2 g/dl, no identified triggering factor for ACS, platelet count > 440 G/l, and PaCO_2_ < 38 mmHg at ACS diagnosis. A PAT-ACS score ≥ 2 yields negative predictive value of 94% [116]. Although this score has not been prospectively evaluated, it seems reasonable to prioritize it over other classic PE scores to assess the pre-test risk of PAT during ACS.

*Paediatrics*: Recent studies reported PAT prevalence of 0.4% [118] and 0.53% [119] among hospitalized children with SCD. Multivariable analysis identified older age, female sex, central venous catheter use, chronic kidney disease, history of stroke, duration of hospitalization, and PICU admission as independent risk factors for PAT [118]. Similarly, patients with PAT in the study by Bala et al. were significantly older (17.4 vs. 10.7 years) [119]. However, no pediatric study has validated the use of thoracic CT angiography to diagnose PAT during ACS in children with SCD. Clinical prediction scores used in adults for PAT (Wells, modified Wells, Geneva) have not been validated in children. Given the limited epidemiological data and the need to minimize radiation exposure and contrast agent use in children, it is advisable to assess the potential benefits of CT on a case-by-case basis in pubertal patients with SCD admitted to PICUs for severe ACS complicated by right ventricular failure evident on echocardiography.

R9: Echocardiography

*R9.1: Adults*: Right ventricular stress should probably be assessed with echocardiography for prognostic evaluation in adult patients with severe ACS. (*Grade 2+, strong agreement*).

*R9.2: Pediatrics*: The experts suggest that in children, right ventricular stress be assessed with echocardiography for prognostic evaluation in pediatric patients with severe ACS (*Expert opinion, Strong Agreement*).

*Rationale*

Chronic pulmonary hypertension (PH) is not negligible in adults with SCD [120] and appears early during the course of the disease [121, 122]. PH is associated with increased morbidity and mortality [123]. However, there are few studies evaluating acute PH during ACS. PH can be assessed by measuring systolic pulmonary artery pressure (via the maximum velocity of tricuspid regurgitation, Vmax TR) and by assessing the consequences of PH on the right ventricle (dilation and septal dyskinesia, defining cor pulmonale).

*Adults*: A single-center prospective study involving 70 adults with severe ACS—defined by at least one of the following criteria: respiratory rate over 30/min, signs of respiratory distress, PaO_2_ in room air below 60 mmHg, respiratory acidosis, altered consciousness, extensive lung opacities, or multiorgan failure—hospitalized in the ICU directly addressed this question [124]. Vmax TR was ≥2.5 m/s in 60% of patients and ≥3 m/s in 37% of patients; 13% had cor pulmonale. Vmax TR ≥ 3 m/s was associated with increased in-hospital mortality [124]. Concerning biomarkers, BNP > 30 pg/ml was the most correlated with Vmax TR ≥ 3 m/s (rho 0.54, *p* < 0.01; AUC 0.83 ± 0.05) [124]. In another study by the same group, 83% of adults with the most severe forms of ACS, i.e., fulfilling the criteria for moderate-to severe Acute Respiratory Distress Syndrome (ARDS) presented acute cor pulmonale (versus 20% in patients with ARDS with other etiologies) [125]. Acute cor pulmonale was associated with increased mortality at Day 28 in patients with ARDS secondary to ACS [125]. These findings are in line with previous reports on ARDS with other etiologies showing an association between RV dysfunction and mortality in adults [126, 127].

*Paediatrics*: No paediatric study has evaluated echocardiographic markers of right ventricular stress in predicting the prognosis of children with SCD presenting with ACS. Two studies [128, 129] have demonstrated a significant association between right ventricular systolic dysfunction, either at admission or during hospitalization, and mortality in children with ARDS, in which ACS can be a potential cause.

R10: Systemic corticosteroids

Systemic corticosteroid therapy should probably not be used routinely for the management of ACS in adults and children with SCD (*Grade 2−, Strong agreement*).

*Rationale*

Patients with SCD experiencing ACS exhibit a severe systemic inflammatory state that increases the expression of endothelial adhesion receptors, worsening microvascular occlusion. Corticosteroids can inhibit pro-inflammatory cytokines and endothelial cell activation, thereby reducing the expression of P-selectin and Vascular Cell Adhesion Molecule-1, which are key adhesion factors on the endothelium.

Patients with ACS experience a severe systemic and pulmonary inflammatory response [130]. Systemic corticosteroid therapy has been suggested to reduce the inflammatory response and intravascular haemolysis during ACS [131]. In a study by Quinn et al., which included only 12 patients (nine children and three adults), corticosteroids were associated with shorter hospitalization duration and a reduced need for blood transfusions [132].

Two key studies have explored the effects of corticosteroids on acute crises, including ACS, in children with SCD. In 1994, Griffin et al. conducted a double-blind RCT on 56 VOC episodes, including 15 ACS episodes, in 36 children with SCD under 21 years old (average age 7.7 years) [74]. Methylprednisolone at 15 mg/kg/day for two days significantly reduced analgesic use duration (41.3 vs. 71.3 h in the placebo group, *p* = 0.03). However, risk of rebound pain led the authors to advise against routine use without further studies.

In 1998, Bernini et al. showed that dexamethasone (4 doses of 0.3 mg/kg every 12 h) significantly improved outcomes in 43 episodes of mild to moderate ACS in 38 children (average age 6.7 years) [133]. Dexamethasone reduced hospital stay, clinical deterioration, need for transfusions, oxygen therapy duration, analgesic use, opioid doses, and persistent fever rates. Despite some readmissions, (6/22 in the dexamethasone group vs. 1/21 in the placebo group, *p* = 0.095, with only one experiencing ACS upon readmission), the study showed corticosteroids to be beneficial for mild to moderate ACS in children with SCD.

A 2011 study by Quinn et al. aimed to validate these findings but was discontinued early due to insufficient recruitment; though reduced hospital stays and a trend towards increased readmissions were observed in the corticosteroid group [132].

Two retrospective studies found no significant effect of corticosteroids on hospital stay, transfusion rates, or readmission rates in children with SCD and ACS [134, 135], but four others linked corticosteroid use to a higher risk of readmission [136–139].

Overall, while corticosteroids may reduce ACS severity in children with SCD, the risk of early rehospitalization necessitates caution, consistent with findings on patients with SCD and immune diseases [140, 141] and regarding outpatient care [139]. A meta-analysis found a higher early readmission rate for sickle cell crises with corticosteroid use (odds ratio 3.36, 95% CI: 1.84–6.15) [142]. While combining corticosteroids with transfusion therapy may help to reduce rebound effects, evidence is still limited [134, 143]. Further research is needed to evaluate the potential benefits of corticosteroids in adults and children with SCD.

R11: Non-invasive ventilation

*R11.1: Adults:* Non-invasive ventilation should probably not be used routinely during ACS in adults with SCD. (*Grade 2-, strong agreement*)

*R11.2: Paediatrics*: The experts suggest testing the efficacy and tolerance of non-invasive ventilation on a case-by-case basis during ACS in children with SCD. (*Expert opinion, strong agreement*).

*Rationale*

The pathophysiology of ACS involves alveolar hypoventilation, bone marrow infarction, atelectasis, and hypoxia. Several authors have proposed adding non-invasive ventilation (NIV) to standard treatment.

*Adults*: The pathophysiology of ACS has led some authors to suggest adding NIV to standard treatment [88]. NIV refers to non-invasive respiratory support with two levels of pressure support. Several studies have mentioned the use of NIV in adults with ACS [144–148].

The only RCT evaluating NIV during ACS involved 71 episodes in 60 adults, randomized into two groups: NIV sessions plus supplemental oxygen versus supplemental oxygen alone [149]. The NIV group showed rapid improvement in respiratory rate and arterial carbon dioxide pressure (PaCO_2_), but there was no improvement in comfort, arterial oxygen pressure (PaO_2_) at Day 3, morphine use, number of transfusions, or length of stay. The study’s limitations included being single-center and potentially underpowered. The NIV dose was low (4 h per day), the severity of ACS was generally mild (some patients had normal chest radiographs, and two-thirds had PaO_2_ ≥ 65 mmHg), and patients in the NIV group were more severe at baseline. Despite these limitations, the physiological benefits of NIV, such as reducing intra-pulmonary shunt and improving gas exchange, could justify its use as a therapeutic test in severe ACS so as to avoid invasive ventilation, which is independently associated with higher in-hospital mortality [150]. NIV testing might be most beneficial for selected patients, particularly those with hypercapnia, which occurs in 20% of ACS cases [149].

*Paediatrics*: Several studies have reported use of NIV for ACS in children with SCD, either in descriptive series of acute respiratory failure that includes ACS [151–153]; or in studies specifically focused on ACS [6, 148, 154–156]. Continuous two-level positive pressure ventilation (Pressure Support, PS) has been used in 53% of patients hospitalized in PICUs for ACS, and up to 71% in units with a high volume of ACS cases [157]. A prospective study involving 30 children with severe ACS admitted over one year to two PICUs with SCD expertise found that continuous PS was well-tolerated and effective, significantly reducing the Clinical Respiratory Score (CRS; > 6 at the start of NIV) within 4 h [158]. However, no RCT has specifically tested the effects of NIV in children with SCD and ACS. A retrospective pediatric study [154] suggested that early NIV use could reduce intubation and transfusion rates, but this study lacked a control group and had a low level of evidence, relying on comparisons to figures in the literature. Another pediatric before-and-after study [87] reported benefits from a protocolized approach that included early use of BiPAP in children with ACS (CRS > 4), but NIV was not independently evaluated.

Despite the lack of robust literature, substantial pediatric experience suggests that two-level positive pressure NIV in severe ACS in children with SCD, especially in units experienced in managing these cases, may reduce intubation rates and potentially decrease the need for red blood cell transfusions. Prospective randomized studies are needed.

R12: Inhaled nitric oxide

High-dose nitric oxide should probably not be used routinely for the management of adult and children with SCD developing VOC or ACS. (*Grade 2-, Strong Agreement*)

*Rationale*

The metabolism of nitric oxide is profoundly altered in SCD (underproduction, impairment of transport to vascular and tissue receptors, scavenging by free plasma haemoglobin secondary to haemolysis) [159]. These alterations can contribute to the pathophysiology of VOC and ACS. Inhaled nitric oxide exerts a pulmonary vasodilator effect in ventilated areas, blunting pulmonary hypertension (which is frequent and severe during ACS) [124], and improving ventilation-perfusion ratios.

*Adults:* In 2011, a prospective multicenter double-blind randomized study compared high-dose inhaled nitric oxide to placebo for at least 72 h in 150 paediatric and adult SCD patients hospitalized for VOC [160]. There was no significant difference between the two groups regarding the primary outcome (resolution of the crisis). One of the secondary outcome measures was the incidence rate of ACS requiring transfusion, and there was no significant difference between the two groups for this criterion: 10.7%, 95% CI: 4.7–19.9) in the inhaled nitric oxide group versus 9.3%, 95% CI: 3.8–18.3) in the placebo group. In 2015, in a prospective multicenter double-blind study against placebo, Maitre et al. tested the effect of high doses of inhaled nitric oxide against an inhaled nitrogen placebo in 100 adult patients with ACS [161]. The primary criterion was the number of patients with treatment failure on Day 3 (composite criterion including death, intubation, decrease in PaO_2_/FiO_2_, new transfusion, or bleeding). There was no difference in the primary outcome between the two groups (46% in the inhaled nitric oxide group versus 58% in the placebo group, OR 0.8 [0.54–1.16]). All secondary criteria were similar between groups. A post hoc analysis suggested an improvement in the subgroup of hypoxemic patients (PaO_2_/FiO_2_ < 300), indicating that the study may not have had enough power.

*Paediatrics*: In a small cohort of 20 SCD patients aged 10 to 21 years with VOC but without ACS [65], inhaled nitric oxide induced a significant decrease in pain intensity evaluated by the visual analog scale. Morphine consumption was significantly lower at 6 h in the inhaled nitric oxide group but not different at H4 and H24. No inhaled nitric oxide toxicity was reported. No publication related to the use of inhaled nitric oxide in Acute Respiratory Distress Syndrome (ARDS) secondary to ACS has been identified in childhood. However, recent international recommendations on pediatric ARDS propose its use in the most severe cases, particularly in cases of demonstrated pulmonary hypertension or right ventricular failure [162], under the framework of rigorous therapeutic testing (efficacy, monitoring, and surveillance of adverse effects). Despite the lack of robust scientific evidence demonstrating the benefit of inhaled nitric oxide in VOC or ACS in children with SCD, it could be tested in cases of ACS managed in PICUs for ARDS with pulmonary hypertension and right ventricular dysfunction.

R13: Therapeutic anticoagulation

Currently available data do not allow a recommendation to be made regarding therapeutic anticoagulation in adult and pediatric patients with ACS without documented deep vein thrombosis or PAT. (No specific recommendation).

*Rationale*

Individuals with SCD have an increased risk of deep vein thrombosis compared to the general population [109, 163]. The occurrence of deep vein thrombosis and/or PE has been identified as a risk factor for mortality in both children and adults with SCD [109, 118]. In adults with SCD, CT with pulmonary angiogram is positive in 17% of cases during ACS [113]. These thromboses in large elastic arteries are often peripheral and fibrinocrural; they are likely to represent the “tip of the iceberg”, and testify to a more extensive in situ thrombotic phenomenon in the pulmonary microvasculature [164]. At the time of guideline scoring, no randomized controlled trials of therapeutic anticoagulation in patients with ACS had yet been published (one trial was completed with results pending: TASC: NCT02580773). There is a single-center retrospective study [165] and a single-center feasibility study [166] of low evidence with small numbers or missing data, precluding any robust interpretation. No study has assessed the role of therapeutic anticoagulation during ACS in children with SCD.

R14: Prophylactic anticoagulation

*R14.1: Adults*: Thromboprophylaxis is indicated in acutely ill medical patients, including those with ACS. (*No specific recommendation*)

*R14.2: Paediatrics*: Prophylactic anticoagulation should probably be used in children with SCD with ACS in the following cases: pubertal onset, a history of deep vein thrombosis, central venous catheterization, COVID-19, or Moya-Moya disease (after an individual assessment of the risk–benefit ratio). (*Grade 2+, Strong Agreement*).

*Rationale*

The population of patients with SCD accumulates classic risk factors for thromboembolism, such as central venous catheter and prolonged immobilization, along with a state of hypercoagulability and chronic inflammation [167]. As a result, patients with SCD have an increased risk of deep vein thrombosis (DVT) compared to the general population [109, 168].

*Adults: *In all acutely ill medical adult patients (including patients with ACS), current guidelines suggest using prophylactic anticoagulation for thromboprophylaxis [169]. Consequently, the panel makes no specific recommendation.

*Paediatrics: *From 2009 to 2015, medical coding data showed 1.7% prevalence of thromboembolic complications among hospitalized children with SCD (10,454 children aged 0–21 years). Multivariable analysis identified older age (15.9 vs. 10.6 years), female sex, central venous catheter, chronic kidney disease, history of stroke, longer hospital stays, and ICU admission as independent risk factors for DVT and pulmonary embolism (PE). DVT occurrence increased median hospitalization duration (8 vs. 3 days, *p* = 0.0001) and number of hospitalizations (8.5 vs. 4, *p* = 0.0001) [118]. Regarding PE, a recent American study reported 0.53% prevalence among hospitalized children with SCD (0–21 years) from 2010 to 2021, translating to 2.07 PEs per 1000 patient-years across 48 hospitals [119]. Median age for PE patients was significantly higher (17.4 vs. 10.7 years). At PE diagnosis, DVT was present in 27.5% and ACS in 57.5% of cases. PE was associated with prolonged hospital stays (median 8 vs. 3 days, *p* < 0.001), and higher ICU admission rates (46.7 vs. 8.9%, *p* < 0.001) and readmission rates (30-day 12.5%, 6-month 50%). Both DVT and PE are linked to increased mortality risk in children with SCD [109, 118]. Despite uncertainties, due to high thromboembolic risks, preventive anticoagulation should be considered in high-risk scenarios such as puberty onset (G2 stage), history of DVT, presence of central venous catheters or COVID-19 infection, or Moya-Moya syndrome, with careful risk–benefit assessment.

R15: Procalcitonin

The panel makes no recommendation on the use of procalcitonin for initiating antibiotic treatment in adult and children with ACS. (*No recommendation, strong agreement*)

*Rationale*

Exposure to antibiotics is extremely high in patients with SCD due to the significant risk of infection associated with hyposplenism. Moreover, bacterial lung infections are recognized as one of the triggers of ACS.

*Adults: *Several adult studies have shown a good diagnostic performance of procalcitonin (PCT) for the diagnosis of bacterial infection during VOC and/or febrile illness in patients with SCD [170–172]. PCT has a valuable role in the diagnosis of bacterial infection with thresholds < 0.5 ng/ml having a good negative predictive value [170], and thresholds > 1 ng/ml [172] or 2 ng/ml [170, 171] having a good positive predictive value. However, a few patients with documented bacterial infections in these series had low PCT levels. Overall, while PCT appears to be useful in confirming bacterial infection, its use alone for initiating antibiotic therapy in patients with ACS does not seem to be recommendable at this time. A prospective before-after monocentric study showed that a PCT-guided strategy to continue or discontinue antibiotics in adults with ACS may reduce antibiotic exposure, with no apparent adverse outcomes [173].

*Paediatrics*: VOC, whether or not accompanied by fever, does not significantly elevate PCT levels in children with SCD [174]. Conversely, a study involving 46 febrile children with SCD, including six with ACS, found that a PCT level > 0.5 ng/ml was the only factor significantly associated with documented bacterial infection [175]. Another study with a limited sample size (69 documented or possible bacterial infections out of 316 patients) reported significant differences in peak PCT values during ACS between patients without and those with documented bacterial infection: median (interquartile range) of 0.3 (0.2–0.5) vs. 4.6 (0.4–25.4), *p* = 0.007, respectively [176]. Therefore, PCT could assist in diagnosis of infections in children with SCD. However, since *Mycoplasma pneumoniae* could be involved in some cases of ACS, PCT levels may not be discriminatory, as they tend to rise only slightly in such situations [177]. Additionally, no pediatric studies have as of yet investigated the usefulness of repeated PCT measurements to guide antibiotic therapy in ACS. Therefore, while PCT appears useful in confirming bacterial infection, its use alone for managing antibiotic therapy in ACS in children with SCD cannot be recommended at this time, either for initiating or for discontinuing antibiotic therapy. Further studies are warranted.

R16: Antibiotics for ACS

*R16.1: Adults*: The experts suggest administering an empirical antibiotic targeting intracellular bacteria in combination with an antibiotic targeting pyogenic bacteria in cases of severe ACS in adults. (*Expert opinion, Strong agreement*).

*R16.2: Paediatrics*: Empirical antibiotic therapy targeting intracellular pathogens should probably be used in combination with an antibiotic targeting pyogenic bacteria in children with ACS. (*Grade 2+, strong agreement*).

*Rationale*

*Adults: *Currently, there are no RCTs studying the effects of antibiotic therapy on intubation or morbidity-mortality in ACS in children or adults with SCD [178]. A large cohort published in 2000 reported infection as the cause of ACS in 38.6% of adults with SCD. The majority of infectious agents were intracellular (39%) [148]. However, more recent work in the era of multiplex genomic testing has shown Polymerase Chain Reaction (PCR) documentation of intracellular bacteria in only 0 to 3% of adults [179, 180]. In adults, the role of intracellular bacteria as an etiological factor in ACS is therefore currently likely minor. However, it seems reasonable to consider combining an antibiotic targeting intracellular pathogens when there are signs of severity (e.g., hypoxemia).

*Paediatrics: *Even though ACS in children with SCD is often associated with viral detection in the respiratory tract [181], the exact role of these viruses remains unclear [182]. A significant reduction in ACS incidence following the introduction of the 13-valent pneumococcal vaccination highlights the potentially key role of *Streptococcus pneumoniae* in ACS among children with SCD [183]. Besides pyogenic organisms, *Mycoplasma pneumoniae* may also be associated with ACS, especially in very young children with SCD [184, 185], although this has not been confirmed in all studies [182]. A retrospective cohort study included 14,480 hospitalizations of 7178 children with SCD aged 0–22 years diagnosed with ACS or pneumonia between January 2010 and December 2016 across 41 pediatric hospitals [186]. Children with SCD treated with guideline-concordant antibiotic therapy (cephalosporin + macrolide, used in 74% of cases) had a reduced risk of ACS within 30 days (OR = 0.71; 95% CI, 0.50–1.00) and reduced sickle cell-related readmissions for any cause (OR = 0.50; 95% CI, 0.39–0.64) [186]. Badaki-Makun et al*.* analyzed the effects of antibiotic treatment on ACS outcomes in children with SCD, reviewing 21,126 visits of 8856 patients aged 11.2 years (6.1 to 16.5 years) from 48 US hospitals between January 2008 and December 2016 [187]. They found that ceftriaxone alone and a combination of ceftriaxone with azithromycin significantly reduced ACS length of stay and 30-day readmission rates (OR = 0.31; 95% CI, 0.27–0.35 and OR = 0.20; 95% CI, 0.17–0.24, respectively) [187]. Despite the lack of robust evidence, retrospective data suggest the importance of bacterial infection in the genesis or prolongation of ACS in children with SCD. Therefore, effective empiric antibiotic therapy targeting both pyogenic and intracellular organisms should be recommended pending definitive studies.

R17. Intravenous beta-lactams in patients with SCD

In adult and pediatric patients with SCD experiencing severe or difficult-to-treat infections, and without renal insufficiency, intravenous beta-lactams should probably be administered using a loading dose followed by continuous administration, or an increased number of doses per day, in order to improve their pharmacokinetics and pharmacodynamics (*Grade 2+, Strong agreement*).

*Rationale*

Patients with acute complications of SCD and with suspicion of infection often receive antibiotic therapy, including a β-lactam. β-lactams are time-dependent antibiotics, and the pharmacokinetic target is plasma concentration above the minimum inhibitory concentration (MIC) for a certain percentage of time between administrations (% f(T) > MIC). For vulnerable populations and severe infections, the target of 100% f(T) > 1–4 × MIC is desirable [188]. While MIC depends on the pathogen, empirical treatment with cefotaxime should consider the pathogen with the highest MIC (2 mg/L according to the European Committee on Antimicrobial Susceptibility Testing, EUCAST). Patients with SCD exhibit glomerular hyperfiltration from an early age (up to a 66% increase in glomerular filtration rate) and urinary concentration disorders related to sickle cell nephropathy [189, 190], which can alter antibiotic pharmacokinetics, as recently reported for vancomycin [191]. Hyperhydration in these patients might exacerbate hyperfiltration. Additionally, acute complications are associated with systemic inflammatory states that increase the volume of distribution. β-lactams are hydrophilic molecules, usually eliminated by the kidneys, except for ceftriaxone, and their concentration is therefore entirely dependent on the previously mentioned phenomena. When a standard dosage is used, there is a risk of underexposure.

*Adults*: Adult patients with SCD often have glomerular hyperfiltration [192], resulting in faster drug elimination. In a prospective cohort of 32 adult patients with SCD empirically treated with β-lactam antibiotic therapy (amoxicillin, with or without clavulanic acid or cefotaxime) for suspected infection during severe ACS in the ICU, the vast majority (84%) presented undetectable (i.e. < 2 mg/L) trough concentrations [193]. A prospective before-after study assessed 60 consecutive episodes of severe ACS in 58 adult patients with SCD (>75% with glomerular hyperfiltration) receiving 60 mg/kg/day cefotaxime during ACS in the ICU [194]. Patients treated with continuous infusion more often had detectable (i.e. ≥2 mg/L) trough concentrations than those receiving intermittent administration: 28 (93%) vs. 5 (16%), *p* < 0.001. Numerous studies have shown that continuous administration of beta-lactams improves pharmacokinetics and pharmacodynamics in severe infections in the general adult population [195]. It is worth noting, however, that this optimization is necessary only in certain situations, such as difficult-to-treat or severe infections.

*Paediatrics: *Two recent pediatric studies described the population pharmacokinetics of cefotaxime in the context of VOC and ACS. Maksoud et al. studied 78 children with SCD receiving cefotaxime at a dose of 200 mg/kg/day in 4 injections to determine volume of distribution and clearance [196]. Cefotaxime clearance was increased, especially in cases of ACS. The pharmacostatistical model suggested that higher dosages are needed to achieve the pharmacokinetic target of 100% f(T) > 1 × MIC: 100 mg/kg every 6 h or 2 g every 6 h for those over 12 years old [196]. Béranger et al. made the same observation in 49 children, including 14 with SCD [197]. The pharmacostatistical model suggested a dosage of 100 mg/kg/day by continuous infusion preceded by a loading dose of 20 mg/kg so as to meet the pharmacokinetic target of 100% f(T) > 4 × MIC. Intermittent administrations could not achieve the target without significantly increasing the daily dose and risking toxicity. It is important to note that these dosage suggestions excluded children under one month of age and those with impaired renal function [197]. To date, there are no in vivo pharmacological validation studies of simulated dosages in the pediatric SCD population. That is why, pending studies on children with SCD, we recommend continuous infusion administration of cefotaxime at a dosage of 100 mg/kg/day preceded by a loading dose of 20 mg/kg, provided that renal function is normal (glomerular filtration rate > 60 ml/min/1.73 m^2^), and therapeutic pharmacological monitoring is always associated.

### Fourth area: transfusion therapy

R18. Transfusion modality

*R18.1. Adults & paediatrics:* In the event of an acute complication warranting a transfusion in an adult or pediatric patient with SCD, the experts suggest prioritizing simple transfusion if the anemia is severe (total haemoglobin < 7 g/dL) and immediately performing an exchange transfusion (manual or automated) in other cases. (*Expert opinion, Strong agreement*).

*R18.2. Paediatrics:* In case of an acute complication warranting a transfusion in a child with SCD, the experts suggest not to exceed a post-procedure hematocrit of 33 ± 3%. (*Expert opinion, Strong agreement*).

*Rationale*

Exchange transfusion removes HbS and provides HbA, leading to HbS dilution without increased blood viscosity. However, exchange transfusion may need a greater number of RBC units and is consequently associated with a theoretical increased risk of alloimmunization and transfusion-related adverse events. Moreover, the exchange transfusion procedure requires specific devices and trained personnel. It may not be straightforward in non-specialized centers and its implementation may take longer than that of simple blood transfusion. Theoretically, compared to manual exchange, automated exchange (erythrocytapheresis) offers (i) improved blood viscosity [198], (ii) greater reduction in HbS [199], and (iii) better correlation between predicted/observed HbS and hematocrit levels at the end of the procedure [200, 201], although these have not been tested in RCTs. Both manual exchange and erythrocytapheresis transiently reduce pro-inflammatory mediators [202], leukocyte, and platelet counts [198, 199]*.*

*Adults*: Only one observational study has compared the prognosis of 20 patients with SCD undergoing one or more exchange transfusions to that of 20 patients with SCD treated with simple blood transfusion [147]. There was no difference in the length of stay between the two groups. Patients treated with exchange transfusions received an average of 10.3 RBC units (±3) compared to 2.4 (±1.2) for patients treated with simple blood transfusion.

*Paediatrics*: Erythrocytapheresis’s increased blood product use is particularly notable in pediatric patients weighing <20 kg or when the machine circuit volume exceeds 15% of blood volume, necessitating blood priming [203]. The machine and circuit must be pediatric-adapted, and isovolemic dilution [204] is contraindicated within 6 months of a stroke [205].

In emergencies, the time it takes for the patient to reach the erythrocytapheresis site (or for the erythrocytapheresis team to reach the patient) is crucial when estimating the time lapse from diagnosis to achieving HbS and hematocrit targets. If erythrocytapheresis access is difficult, manual exchange remains a viable option [206]. When both techniques are available, the time commitment of each must be weighed against the clinical context [207, 208].

Although erythrocytapheresis can theoretically be performed with a good peripheral venous line, pediatric cases often require a central venous catheter for uninterrupted procedures, commonly seen in manual exchange [201]. Central venous catheter-related complications are common, even when removal is performed immediately after the procedure [209], but can be minimized with proper management [210]. Anaesthetic precautions are essential for children with SCD, particularly with Moya-Moya. Though rare, significant hemodynamic events must be considered in both manual exchange and erythrocytapheresis [201] [199]. Citrate regional anticoagulation is contraindicated in haemorrhagic stroke or high risk of haemorrhagic transformation [201] [205].

R19: Indications for blood transfusion

R19.1: VOC

The experts suggest against the systematic use of transfusion therapy for uncomplicated VOC in adult and pediatric patients with SCD (*Expert opinion, strong agreement*).

*Rationale*

Although chronic transfusion therapy reduces the frequency of hospitalizations for VOC [211], the clinical efficacy of blood transfusion for acute VOC has not been adequately studied. Only one pilot randomized study [212] has evaluated the efficacy of blood transfusion in acute VOC in adult and pediatric patients with SCD. This study had a small sample size (n = 10), and the patients were mildly anaemic (Hb = 8.4 g/dl), receiving step 3 analgesics. The results did not show any benefit of blood transfusion in reducing hospitalization duration, opioid consumption, or pain scores. Two reviews [213, 214] recommend against the systematic use of blood transfusion for acute VOC in patients with SCD. Experts propose blood transfusion based on case-by-case evaluation, for example in the event of severe (<7 g/dL) and/or poorly tolerated anemia or if pain is not controlled despite appropriate analgesics.

R19.2-3: ACS

*R19.2: Adults:* The experts suggest performing a transfusion therapy for the management of ACS in adult patients with SCD who meet severity criteria with regard to hypoxemia (need for oxygen therapy to achieve transcutaneous oxygen saturation of 95%), severe pulmonary hypertension (tricuspid regurgitation velocity > 3 m/s or acute cor pulmonale), or any associated organ failure. (*Expert opinion, Strong agreement*).

*R19.3: Paediatrics:* The experts suggest performing a transfusion therapy for the management of ACS in pediatric patients with SCD who meet severity criteria, with regard to rapidly worsening hypoxemia, or any associated organ failure. (*Expert opinion, Strong agreement*).

*Rationale*

*Adults*: By analogy with VOC, the expected benefit of blood transfusion during ACS is to dilute the level of sickle haemoglobin and limit tissue hypoxia. In the case of hypoxemia, improving oxygenation in a patient with respiratory distress could also limit morbidity.

The recurrence of ACS and its severity in childhood could contribute to the development of chronic lung lesions in the long term [215]. Several observational studies have highlighted favourable effects with blood transfusion during ACS in adult patients with SCD, including reductions in hospital stay [216] and 30-day readmission rate [217].

According to the Cochrane meta-analysis [218], no study provides conclusive evidence on the benefit of blood transfusions in ACS treatment. The decision to transfuse should be based on clinical experience and individual circumstances. Nevertheless, the benefit-risk balance appears to favour transfusion in severe ACS, and there is a consensus among numerous medical societies (UK, USA, Europe) to recommend transfusion (blood transfusion or ET) in this setting.

*Paediatrics*: Since no evidence-based recommendation is possible for transfusion in ACS, previous immunisation must be taken into consideration in the risk–benefit balance evaluation of transfusion for ACS patients, especially if the relevant severity criteria are not met.

While the experts agree that transfusion therapy for mild or moderate ACS in paediatric patients is not mandatory, patients who are not transfused for their ACS episode need to be closely monitored in wards with attested expertise in SCD patient management. As indicated above, evolution towards severe ACS requires transfusion therapy.

If exchange transfusion is indicated for ACS with no associated organ failure, experts have no recommendation regarding exchange technique (automated or manual).

When transfusion therapy is indicated, experts suggest simple transfusion in case of significant decrease of haemoglobin from patient’s baseline and transfusion exchange in all other cases with targeted HbS < 30% and haematocrit 30% (±3%).

R19.4: Stroke

The experts suggest urgent transfusion therapy, ideally within two hours of symptom onset, for adult and paediatric patients with SCD showing signs of stroke. Automated exchange transfusion should be prioritized if time allows, or manual exchange if there are delays in accessing automated techniques. In cases where haemoglobin levels are below 7 g/dl, a simple transfusion is suggested while awaiting exchange transfusion to reach haemoglobin S (or S + C) below 30% with haematocrit 30 ± 3%.

Haemorrhagic stroke may also require exchange transfusion or simple transfusion, especially when neurosurgical procedures are indicated. (Expert opinion, Strong agreement)

*Rationale*

*Adults*: Most often ischemic, stroke remains one of the most frequent causes of morbidity and mortality in patients with SCD. It is associated with circumferential stenosis of intracerebral and cervical carotid arteries. Most of the literature data concern the benefit of blood transfusion programs in primary or secondary prevention of stroke, demonstrating a 92% risk reduction when HbS is maintained <30%. Only one retrospective study in children with SCD [219] has shown a benefit of exchange transfusion vs. blood transfusion in preventing early recurrence of a first stroke. No study has explored the usefulness of blood transfusion in acute stroke in adults.

*Paediatrics*: In cases of suspected stroke in children with SCD, an emergent transfusion therapy should be administered within two hours of symptom onset so as to rapidly reduce HbS levels to below 30% [220–222]. This goal can be achieved in an emergency situation through a blood transfusion if the hemoglobin level is <7 g/dL, with a target hemoglobin level not exceeding 10 g/dL, while awaiting an exchange transfusion. In an ideal situation, it is recommended to use automated exchange transfusion as the first-line treatment, when possible and without delay, the objective being to minimize acute changes in cerebral blood flow, which are considered crucial for reducing neurological damage. Although automated exchange can be quicker to perform than manual exchange [201, 207], the time required to reach a center capable of performing apheresis may be lengthy, significantly increasing the total time between stroke diagnosis and the attainment of HbS and hematocrit goals. Manual exchange, which is more widely available in many hospitals, could therefore be considered based on an individual risk-to-benefit ratio [206].

Regardless of the exchange transfusion techniques used, hemodynamic tolerance must be closely monitored from the beginning of procedural anesthesia for central line insertion until completion of the exchange transfusion procedure, particularly in patients with Moya-Moya disorder, as approximately 10% of patients may experience elevated heart rates or drops in arterial pressure [195, 197]. For low-weight patients, i.e., those below 20 kg, or when the priming volume of the circuit exceeds 15% of the patient’s estimated blood volume, automated exchange transfusion requires specific management, such as priming with blood and manually setting the exchange machine parameters [203]. The technique of isovolemic dilution [204], designed to conserve blood products during exchange, is considered dangerous within six months of an acute stroke [205]. Additionally, citrate anticoagulation for automated exchange is contraindicated in cases of hemorrhagic stroke or ischemic stroke with a high risk of hemorrhagic transformation [201] [205].

For the rare SC patients presenting with signs of an acute stroke, blood exchange should be considered, as blood viscosity plays a critical role in stroke pathophysiology. The hemoglobin S + C fraction must be reduced to below 30% by the end of the procedure.

The different exchange techniques used in emergency settings appear to entail comparable blood product usage and risk of delayed hemolytic transfusion reactions (DHTR) [199, 201, 203, 204, 207, 208, 223–226].

R19.5: Priapism

The experts suggest transfusion therapy for persistent acute ischemic priapism in adult and paediatric patients with SCD when non-invasive and invasive treatments (aspiration and/or intracavernosal injection) fail or when priapism has been ongoing for at least four hours. (*Expert opinion, Strong agreement*).

*Rationale*

The initial treatment for priapism in patients with SCD involves non-invasive and invasive measures outside the scope of transfusion (see R22). Two contradictory observational studies exist regarding the potential benefit of exchange transfusions in cases of priapism in adults with SCD. McCarthy et al. [227] found no benefit on pain or detumescence (small sample size, n = 7) during the acute phase, whereas Ballas et al. [228] suggested a benefit in preventing recurrences in chronic cases. Case reports, such as those by Siegel et al. [229], have noted neurological symptoms (headaches, seizures, altered consciousness) during transfusions for priapism, warranting case-by-case evaluation. If there is no improvement after four hours, transfusion may be considered, although the evidence remains insufficient.

R19.6: Splenic sequestration

The experts suggest transfusion therapy in cases of splenic sequestration in adult and paediatric patients with SCD. (*Expert opinion, Strong agreement*)

*Rationale*

Acute splenic sequestration is defined by a decrease of at least 2 g/dL (or 20%) from baseline haemoglobin levels, accompanied by an increase in spleen volume (splenic overflow) of at least 2 cm, with a normal or elevated reticulocyte count [223]

There are no clinical studies comparing outcomes with or without blood transfusion in acute splenic sequestration. However, there is unanimous consensus among experts [81, 81, 230] that acute splenic sequestration is a medical emergency requiring blood transfusion. Higher-than-expected transfusion yields because of release of sequestrated red cells justify caution regarding the amount of packed red blood cells transfused, the objective being to avoid post-transfusion haematocrit >35%. The major risk of acute anaemia and hypovolemia in SCD patients with acute splenic sequestration justifies urgent implementation of blood transfusion.

R19.7: Acute organ failure

The experts suggest transfusion therapy in the event of acute organ failure. (*Expert opinion, Strong agreement*)

*Rationale*

Some case reports or small series suggest the benefits of simple blood transfusion or exchange transfusionin cases of organ failure directly related to SCD (e.g., acute hepatic VOC) [225, 226] or not directly related (e.g., sepsis). Organ failures are associated with numerous factors that may promote HbS polymerization, such as acidosis and hypoxia. To mitigate these effects, a blood transfusion procedure may be considered.

R19.8: Delayed haemolytic transfusion reaction (DHTR)

Transfusion therapy should probably not be performed in adult and pediatric patients with SCD presenting with Delayed Haemolytic Transfusion Reaction. (*Grade 2-, Strong Agreement*)

*Rationale*

Driven by complex immune mechanisms, DHTR involves the rapid destruction of transfused red blood cells in a patient with SCD. In adults, transfusion therapy increases mortality in patients with DHTR [231, 232].

Two recent studies reported 78 episodes since 2005 of DHTR in a French cohort of children with SCD [233, 234]. All children with SCD retransfused in the context of DHTR without prior immunosuppressive treatment experienced a recurrence, at times severe, of haemolysis.

If an RBC transfusion is deemed necessary during an acute episode of DHTR due to poor clinical or biological tolerance of anaemia (e.g., total Hb < 3 g/dL and/or organ failures), it should be carried out after consultation with a paediatric or adult expert center of SCD and may have to be accompanied by immunological therapies (e.g., corticosteroids, immunoglobulins, Eculizumab, Rituximab). Erythropoietin should also be considered in case of reticulocytopenia.

R20: Diagnosis of DHTR

R20.1-2. Nomogram

*R20.1: Adults:* The Mekontso Dessap nomogram should probably be used to diagnose DHTR in adult patients with SCD (*Grade 2+, Strong agreement*)

*R20.2: Paediatrics.* The experts suggest using the Mekontso Dessap nomogram to diagnose DHTR in children with SCD (*Expert opinion, Strong agreement*)

R20.3. The experts suggest systematically performing a complete blood count and haemoglobin electrophoresis within 48 h following RBC transfusion in adult and paediatric patients with SCD at risk of DHTR. (*Expert opinion, Strong agreement*).

*Rationale*

DHTR poses a life-threatening risk, particularly when diagnosis and management are delayed [231]. The definition of DHTR first proposed by Petz et al. [235] is based on empirical observation of haemoglobin levels dropping below pre-transfusion levels, accompanied by markers of haemolysis and vaso-occlusive complications such as VOC. However, this definition does not account for the post-transfusion disappearance of HbA, a form of haemoglobin absent in patients with SCD (except for those with S beta + thalassemia) and introduced only through transfusions. In the international literature, a common reference for DHTR is a decrease in HbA to less than 50%, although no specific time frame post-transfusion is typically defined.

*Adults: *The Mekontso Dessap nomogram (available at https://reamondor.aphp.fr/nomogram-2/) is a diagnostic tool for DHTR, based on the disappearance of HbA mass rather than simply a decrease in its percentage, serving as a biological marker of transfused RBC survival [236]. It requires four elements: the percentage of HbA and the concentration of haemoglobin (g/dl) both post-transfusion (within 48 h) and later, when DHTR is suspected.

The only study utilizing this nomogram involved 58 DHTR cases that received preventive Rituximab treatment [237]. Although the nomogram lacks formal evaluation, it is reasonable to recommend measurement of post-transfusion total Hb and HbA, particularly in patients at risk of DHTR, the objective being to facilitate timely diagnosis [232]. Repeating these measurements one month later may also help to diagnose low-grade DHTR, along with erythrocyte antibody identification in view of detecting alloimmunization.

*Paediatrics*: Many cases of DHTR have been reported in children with SCD, of which the frequency is estimated at 5% in recent French studies [234]. There are currently no studies on use of the Mekontso Dessap nomogram in pediatric patients with SCD. For the few children reported in the literature for whom the nomogram can be retrospectively plotted, it strongly supports the diagnosis of DHTR [233, 238]. Pending its definitive validation in paediatrics, experts recommend use of the Mekontso Dessap nomogram in children with SCD with DHTR suspicion, especially those with DHTR risk factors, i.e., in case of history of DHTR or alloimmunization, and in patients who have received fewer than 12 RBC transfusions.

R21. Prediction of DHTR

*R21.1: Adults:* The experts suggest using the Narbey predictive score to assess the risk of DHTR before transfusion in adult patients with SCD. They suggest categorizing the risk as: i) not significant if the score is less than 8; and ii) significant if the score equals or exceeds 8, or if the patient has a history of confirmed DHTR. (*Expert opinion, strong agreement*).

*R21.2: Paediatrics:* The Narbey predictive score should probably not be used in children with SCD to assess the risk of DHTR. (*Grade 2-, Strong consensus*)

*Rationale*

DHTR incidence is increasing in patients with SCD and can be life-threatening [231, 239, 240], particularly when diagnosis and management are delayed. DHTR primarily occurs in patients having received transfusions in acute settings.

*Adults: *A prospective study identified three key risk factors for DHTR in adults with SCD: a history of alloimmunization (positive irregular agglutinin screening, past or present), a history of DHTR (regardless of severity), and a limited number of blood transfusions (fewer than 13 in the cited study) [239]. These parameters were incorporated into a multivariable analysis to develop a risk score for DHTR in patients requiring blood transfusion for acute complications. The score assigns 5 points for a history of DHTR, 6 points for alloimmunization status, and 8 points for fewer than 13 transfusions. Risk categories are defined as low (<8), intermediate (8–14), and high (>14). For high-risk patients, transfusion should be reconsidered if the acute complication does not pose an immediate threat to life. In these cases, extended phenotype-matched red blood cells (FY, JK, MNS) and immunosuppressive therapy (e.g., Rituximab) are recommended. Despite the score’s usefulness, a history of DHTR is particularly critical and may have been underestimated in the original study due to bias; patients with a history of DHTR were seldom retransfused, potentially underestimating their risk [238]. When considering transfusion in an adult with SCD, a score of 8 or higher, or a confirmed history of DHTR, indicates significant risk. In such cases, transfusion decisions should be carefully reconsidered, and alternative treatments explored. If transfusion is still deemed necessary despite high risk, consultation with an expert center is recommended.

*Paediatrics*: The predictive score for DHTR proposed by Narbey et al. [239] has not been validated in pediatric patients, and its retrospective application to pediatric DHTR cases often results in intermediate risk values, making it unable to effectively discriminate between cases [233, 234, 238, 241–244]. While the components of the score are relevant for assessing transfusion risk, its weighting does not appear reliable in children. In two recent paediatric studies [233, 234]) involving 78 children with SCD and DHTR, 72 presented with at least one of the three risk factors included in the Narbey score: a history of DHTR, a history of alloimmunization, or fewer than 12 RBC transfusions. The majority of cases (68/78) occurred after an occasional RBC transfusion, usually for a VOC. Therefore, there is currently no validated score specific to paediatrics for predicting transfusion risk in children with SCD.

### Fifth area: priapism

*R22.1: Adults:* In cases of persistent, painful erection in an adult with SCD suggesting acute ischemic priapism, experts recommend promptly initiating non-invasive measures (such as massages, physical exertion, and possibly oral alpha-adrenergic agonists). If priapism lasts for more than an hour, an intracavernosal injection of alpha-adrenergic agonists should be considered, with a repeat injection after 20 min if the first is ineffective, the objective being to minimize complications and long-term sequelae. (*Expert opinion, Strong agreement*).

*R22.2: Adults:* For ischemic priapism persisting for four hours or more in adults with SCD, experts recommend aspirating cavernosal blood, followed by an intracavernous injection of alpha-adrenergic agonists. These steps should be repeated multiple times before considering surgical intervention, the objective being to minimize complications and long-term sequelae. (*Expert opinion, Strong agreement*).

*R22.3: Paediatrics:* Experts suggest performing an intracavernous injection of alpha-adrenergic agonists and draining the corpora cavernosa in a child with SCD who has been experiencing acute priapism for more than an hour. (*Expert opinion, Strong agreement*).

*Rationale*

**Fig. 3:**
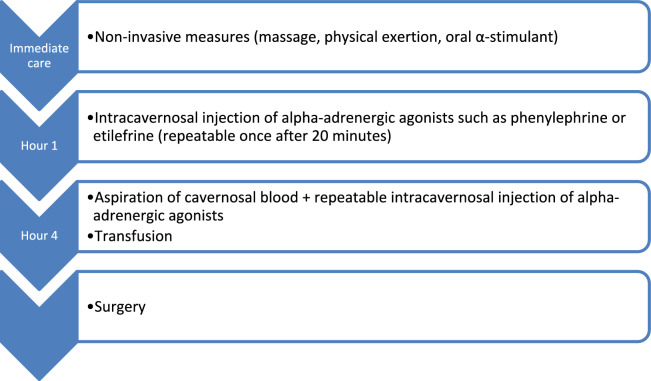
Management of a priapism in adults with sickle cell disease

Figure 3 summarizes the proposed management of priapism in adults with SCD. Priapism is defined as a prolonged erection without sexual stimulation. There are three types: ischemic priapism (the most common, painful due to venous obstruction and low flow), intermittent priapism (recurrent and transient), and non-ischemic priapism (painless, caused by arterial blood influx and high flow). Ischemic priapism can lead to tissue hypoxia, acidosis, and irreversible erectile dysfunction. Recovery of erectile function depends on the duration of the priapism and time to treatment. Prior episodes of priapism also worsen long-term function. There are no high-quality randomized controlled trials on acute priapism treatment in SCD or the general population [245], which is why management relies on urological expert guidelines [246–249].

*Adults: *Adults with SCD of SS genotype experience ischemic priapism in 35–48% of cases during their lifetime. The time of erection onset should be documented. Ischemic priapism should be considered as an emergency [246, 248–250] and treatment is aimed at achieving rapid detumescence so as to prevent permanent damage, combining general measures and local interventions (aspiration and injection).

General measures, including oxygen therapy (aimed at achieving transcutaneous oxygen saturation of 95%), analgesics, intravenous hydration, physical activity [248, 249, 251] and possibly oral alpha-adrenergic agonists can be started while awaiting urological management. However, none of these measures have proven effective in the treatment of acute ischemic priapism [206, 250, 252, 253]. Only 0 to 37% of priapism cases in patients with SCD have been resolved with systemic treatment alone, while much better outcomes have been observed with local interventions [246, 247, 249].

Local interventions include cavernous aspiration, possibly combined with saline irrigation [247–254]. Drainage should continue until oxygenated red blood appears, allowing sufficient time for complete drainage, which can take 20–30 min [249–251]. Aspiration allows for decompression of the corpora cavernosa, promotes restoration of intracavernous blood flow, reduces pain, and decreases local anoxia and acidosis [248, 249]. This procedure is usually performed by a urologist in the emergency room and requires a penile block with local anesthesia (e.g., 1% lidocaine). Aspiration alone is effective in 30% of cases [247, 250, 254].

Alpha-adrenergic agonists such as phenylephrine or etilefrine can be injected intracavernously [247, 248, 250, 254, 255]. The success rate of sympathomimetic injection after cavernous aspiration ranges from 43 to 95% [246, 247, 256–258].

Other options include ephedrine, epinephrine, norepinephrine, and metaraminol [249, 250]. Injections must be carefully monitored due to the risk of hypertension and are contraindicated in patients with a history of stroke, closed-angle glaucoma, coronary insufficiency, hyperthyroidism, or poorly controlled hypertension.

When priapism persists despite general measures or for more than one hour and less than four hours, the French Urology Association recommends starting with an intracavernous injection of etilefrine or phenylephrine, prior to any cavernous aspiration, and repeating the injection after 20 min if ineffective [248].

Surgical treatment should only be considered if these repeated measures fail or if priapism lasts more than 48 h [248]. The reference surgical procedure is caverno-spongy anastomosis, though it is less often needed in patients with SCD compared to the general population [259].

*Paediatrics: *Prevalence of priapism in pediatric patients with SCD ranges from 2 to 6%. The recommended treatment for ischemic priapism in children with SCD is penile decompression by needle aspiration, followed by injection of alpha-adrenergic agonists medications such as etilefrine [249, 260–264]. This treatment is indicated for priapism persisting for more than one hour. Several small case series have demonstrated the potential role of intracavernous injection of etilefrine followed by drainage of the corpus cavernosum in relieving acute priapism in emergency settings [265]. An RCT comparing etilefrine to placebo over a duration ranging from two weeks to six months in an outpatient setting did not measure detumescence [266]. Due to the risk of bias in this trial and the small number of participants, authors of the Cochrane review [245] considered evidence certainty to be low to very low and stressed the need for well-designed, adequately powered multicenter RCTs to evaluate the efficacy of specific interventions for priapism in SCD. In the absence of robust data but considering the experience of expert centers, it seems reasonable to consider intracavernous injection of etilefrine and drainage of the corpus cavernosum in children with SCD presenting with acute priapism lasting more than one hour. If there is no improvement after four hours, transfusion therapy could be considered, although the level of evidence is likewise insufficient.

## Supplementary Information

Additional file 1.

## Data Availability

Not applicable.
