# Supplementary material for: Guidelines for the management of emergencies and critical illness in pediatric and adult patients with sickle cell disease
Source: Ann Intensive Care. 2025 May 29;15:74. doi: 10.1186/s13613-025-01479-3 (PMC12123041; doi:10.1186/s13613-025-01479-3)
Supplement: Supplementary file 1 — Additional file 1. [file 13613_2025_1479_MOESM1_ESM.docx]

### Table S1: Recommendations according to the GRADE methodology

|  | Recommendations according to the GRADE methodology | |
| --- | --- | --- |
| High level of proof | Strong recommendation   “…should be done…” | Grade 1 + |
| Moderate level of proof | Optional recommendation   “… should probably be done…” | Grade 2 + |
| Insufficient level of proof | Recommendation in the form of an expert opinion | Expert opinion |
|  | “The experts suggest…” |  |
| Moderate level of proof | Optional recommendation   “… should probably not be done…” | Grade 2 − |
| High level of proof | Strong recommendation   “…should not be done…” | Grade 1 − |
| Insufficient level of proof |  | No recommendation |
